# Neurovascular mechanisms of high-altitude retinopathy and the potential therapeutic relevance of natural compounds

**DOI:** 10.3389/fcell.2026.2001737

**Published:** 2026-10-28

**Authors:** Shuoyu Yue, Ruijie Shi, Yingying He, Ju Huang, Xiangjie Leng, Yanxia Wu, Jing Huang, Mingxu Zhang, Xuejing Lu

**Affiliations:** 1 Chengdu University of Traditional Chinese Medicine, Chengdu, China; 2 Affiliated Hospital of Chengdu University of Traditional Chinese Medicine, Chengdu, China

**Keywords:** blood-retinal barrier, electroretinography, high-altitude retinopathy, hypobaric hypoxia, natural products, OCTA, retinal neurovascular unit, salidroside

## Abstract

High-altitude retinopathy (HAR) is usually described by fundus changes after ascent. That definition misses the biological problem that makes HAR worth studying: hypobaric hypoxia stresses two separate ocular circulations at once, along with a metabolically demanding neural tissue and a blood–retinal barrier (BRB) that maintains fluid balance. The response may stay a reversible vascular adjustment, or it may progress to hemorrhage, macular injury, and visual dysfunction. Optical coherence tomography angiography (OCTA), functional OCT and electroretinography (ERG) show that vascular and neural response scan diverge: perfusion-related compensation may persist while retinal function declines, and the two may recover on different time scales after reoxygenation. The high-altitude retinal response is therefore better viewed as an adaptation–mismatch–decompensation spectrum than as a linear cascade from hypoxia to cell death. In this Review, reversible vascular or metabolic adjustment without barrier failure, structural injury or persistent functional loss is termed physiological adaptation; HAR is reserved for pathological retinal findings attributable to altitude. This Review examines HAR at three linked levels: clinical differential diagnosis, the retinal neurovascular unit, and the potential therapeutic relevance of natural compounds and extracts. We distinguish reversible vascular adjustment from neurovascular mismatch, barrier failure and regulated cell death. We summarize current medical management without extrapolating general altitude-illness drugs to unproven retinal indications, and we assess chemically defined natural compounds and extracts using a defined evidence hierarchy. Salidroside from a well-characterized Rhodiola tangutica extract, resveratrol, and hesperidin have direct high-altitude retinal data, but the evidence remains preclinical. Studies in other retinal hypoxia or ischemia models point to mechanisms involving mitochondrial quality control, ferroptosis, NLRP3 signaling, endothelial junctions, and glial metabolism; they do not establish efficacy in HAR. Progress will require longitudinal multimodal human phenotyping, standardized hypobaric models, retinal pharmacokinetics, causal target engagement and trials designed around exposure, susceptibility, and recovery.

## Key points

The high-altitude retinal response runs from physiological adaptation through neurovascular mismatch and decompensated injury. HAR should be reserved pathological findings—barrier leakage, hemorrhage, edema, focal structural injury, or reproducible functional loss—not for every reversible vascular change.

OCTA, functional OCT and ERG show that vascular structure, perfusion, and neural function can change on different time scales during exposure and recovery.

The retinal neurovascular unit is a useful framework than any isolated pathway. Endothelial cells, pericytes, Müller glia, astrocytes, microglia, retinal ganglion cells and photoreceptors exchange metabolic and inflammatory signals.

Descent, oxygen, and management of concomitant altitude illness remain the clinically defensible core. Acetazolamide and dexamethasone are not established HAR-specific retinal treatments.

Salidroside, a chemically characterized Rhodiola tangutica extract, resveratrol, and hesperidin have direct animal evidence, but no natural product has demonstrated human efficacy for HAR.

Developing natural products for HAR requires botanical authentication, chemical fingerprints, measurements of active exposure, retinal pharmacokinetics, target engagement, and functional outcomes—just another list of altered proteins.

## 1 Methods and evidence approach

This is a structured narrative review, not a systematic review or meta-analysis, and we do not claim their methods or completeness. We searched PubMed-indexed Europe PMC through 23 August 2026 using title/abstract queries, followed by targeted searches for HAR, high-altitude ocular imaging, hypobaric retinal injury, neurovascular-unit biology, altitude-illness management, salidroside and Rhodiola, resveratrol, hesperidin, and selected natural compounds. We prioritized direct human HAR and high-altitude retinal physiology studies, then hypobaric animal models, then other retinal hypoxia/ischemia models. Each cited record was checked against PubMed for PMID, normalized title, and DOI; one older record without a PubMed DOI was also checked against Crossref. Reference identifiers were cross-checked in PubMed and, where needed, Crossref.

Purified-compound studies are not evidence for the source plant, and extract studies cannot be attributed to a single constituent without fractionation, exposure, or causal experiments. The evidence hierarchy is deliberately conservative: findings from other retinal diseases can suggest mechanisms for testing but cannot establish HAR prevention or treatment. In the mechanistic sections, we identify direct HAR or high-altitude retinal findings explicitly and label evidence from other retinal diseases, systemic hypoxia, or cell models as supporting or extrapolated.

## 2 From visible fundus signs to a dynamic neurovascular disorder

High altitude lowers inspired oxygen pressure without changing the fraction of oxygen in air. The immediate physiological response is systemic—hyperventilation, altered acid–base balance, sympathetic activation and changes in cardiac output—but tissue survival depends on local regulation (Han et al., 2024). The retina is an unusually demanding test of that regulation. Photoreceptors consume large amounts of oxygen, retinal ganglion cells (RGCs) maintain long axons and continuous signaling, and the tissue must preserve optical transparency while restricting fluid and solute entry. Its inner layers are supplied by the retinal circulation; the outer retina relies mainly on the choroid. These circulations differ in flow, oxygen extraction, innervation and autoregulatory capacity. A fall in arterial oxygen therefore creates a spatially uneven retinal exposure (Arjamaa and Nikinmaa, 2006; Kaur et al., 2008; Pournaras et al., 2008; Kur et al., 2012; Newman, 2013; Linsenmeier and Zhang, 2017; O'Leary and Campbell, 2023).

HAR was defined clinically long before current imaging could separate vascular, neural and barrier responses. Classic expedition studies described venous engorgement, tortuosity, disc hyperemia and retinal hemorrhage, often in people who retained useful vision (McFadden et al., 1981; Wiedman and Tabin, 1986; Wiedman and Tabin, 1999; Müllner-Eidenböck et al., 2000). Later work added fluorescein leakage, OCT-defined retinal thickening, layer-specific OCTA measurements, ERG changes and choroidal flow. The added measurements change the question from a catalog of signs to a description of process. A photograph taken at one altitude shows phenotype; repeated vascular and electrophysiological measurements show course. Recent human studies show that capillary or choroidal compensation can coexist with declining neural function, and that recovery can display hysteresis rather than a simple reversal of ascent (Westwood et al., 2024; De Abreu et al., 2025; Li et al., 2025a; Shi et al., 2025; Paillisser et al., 2026; Yu et al., 2026a; Yu et al., 2026b).

This distinction matters because the word “retinopathy” can imply established tissue damage. At altitude, some visible changes are physiological adjustments. Dilated veins or altered capillary density may reflect increased oxygen delivery, reduced vascular resistance or changes in carbon dioxide. They are not equivalent to BRB disruption. Conversely, preserved acuity does not exclude inner-retinal dysfunction or localized photoreceptor injury. A 2026 case of isolated ellipsoid-zone loss illustrates how outer-retinal damage may be missed if evaluation stops at acuity and ophthalmoscopy (Bailey et al., 2026). The clinical task is therefore to identify where an individual lies between successful adaptation and decompensation, and whether the trajectory is improving or worsening.

Operationally, we use physiological adaptation for reversible vascular or metabolic change without symptoms, BRB leakage, structural lesions or persistent functional deficit. Neurovascular mismatch denotes discordance between perfusion-related compensation and neural or barrier function; it is a mechanistic state, not by itself a clinical diagnosis. HAR denotes altitude-attributable retinal pathology, including hemorrhage, edema or leakage, disc, macular or outer-retinal injury, vascular occlusion or reproducible retinal dysfunction. Borderline cases should be reported by domain and trajectory rather than forced into a binary label.

HAR also sits awkwardly beside acute mountain sickness (AMS) and high-altitude cerebral edema (HACE). The retina and brain share barrier-forming endothelium, glial support and principles of neurovascular coupling, which makes the eye an attractive window on central responses. Yet shared biology does not make retinal findings diagnostic of cerebral disease. Optic-disc swelling has not correlated consistently with AMS, and retinal hemorrhage can occur without neurological deterioration (Wiedman and Tabin, 1999; Willmann et al., 2011; Bosch et al., 2012). Severe retinopathy can accompany brain edema, as recent case evidence again shows, but coexistence does not establish that one is a surrogate for the other (Zając et al., 2026). Neurological symptoms, ataxia or altered mental status must be assessed on their own clinical grounds.

We propose HAR as a failure of retinal neurovascular adaptation. Early compensation changes caliber, perfusion, metabolism and glial buffering. Decompensation begins when these responses no longer match neural demand or preserve compartmental homeostasis. BRB leakage, hemorrhage, edema, mitochondrial injury and inflammatory cell death are consequences of that mismatch, but the order and dominant cell type vary with altitude, time, exercise, hematocrit and susceptibility. This model does not force observations into a universal sequence; it places each within a time-dependent system.

Relative to the 2024 overview by Han et al. (Han et al., 2024), this Review extends the evidence window through August 2026 and adds four elements: a neurovascular-unit framework linking vascular, glial and neuronal responses; an operational distinction between physiological adaptation, mismatch and injury; integration of recent cell- and pathway-level hypobaric studies; and a translational hierarchy for natural compounds that separates direct high-altitude retinal evidence from supporting retinal models (Figure 1).

**FIGURE 1:**
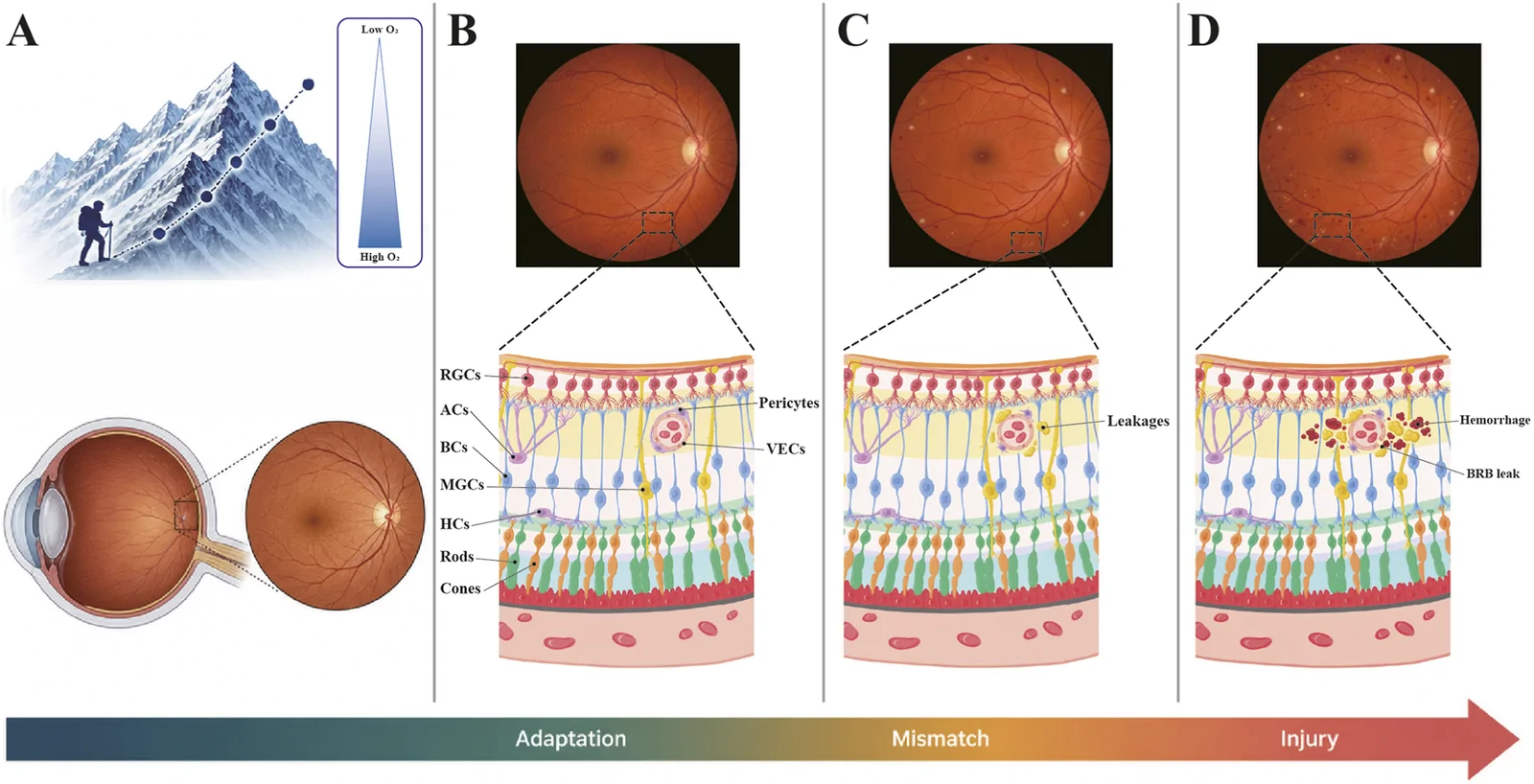
Conceptual adaptation-to-injury continuum in HAR. **(A)** Ascent is accompanied by a progressive reduction in inspired oxygen partial pressure. The ocular schematic indicates the retina and fundus represented in the subsequent panels. **(B)** During compensated adaptation, the fundus remains grossly normal and the retinal neurovascular unit retains organized neuronal layers and an intact endothelial cell–pericyte interface. **(C)** Neurovascular mismatch develops when vascular adaptation no longer meets retinal metabolic demand, resulting in focal endothelial dysfunction and early BRB leakage with limited peripheral fundus abnormalities. **(D)** Decompensated injury is characterized by more extensive BRB disruption, vascular leakage and intraretinal hemorrhage, accompanied by an increased burden of peripheral retinal lesions. The lower arrow summarizes progression from adaptation through mismatch to injury. These states may overlap and are not intended to represent a validated clinical grading system. RGCs, Retinal ganglion cells; ACs,Amacrine cells; BCs,Bipolar cells; MGCs, Müller glial cells; HGs,Horizontal cells; VECs, vascular endothelial cells; BRB, blood–retinal barrier.

## 3 Clinical expression across exposure, tissue compartment and time

### 3.1 A severity spectrum

The common fundus phenotype includes venous dilation and tortuosity, disc hyperemia or swelling, and intraretinal hemorrhages. Cotton-wool spots, macular or premacular hemorrhage, vitreous hemorrhage, vascular occlusion and focal outer-retinal injury are less common but more clinically significant (Barthelmes et al., 2011; Willmann et al., 2011; Han et al., 2024; Rana et al., 2025; Smith et al., 2025; Bailey et al., 2026; Zając et al., 2026). The location of a hemorrhage matters. A small peripheral lesion can be incidental to visual function, whereas a foveal, subhyaloid or vitreous hemorrhage may produce an abrupt deficit. Similarly, OCT thickening around the disc can arise from vascular filling, axoplasmic stasis, cellular swelling or extracellular fluid; the same numerical increase does not have one biological meaning (McFadden et al., 1981; Wiedman and Tabin, 1986; Wiedman and Tabin, 1999; Müllner-Eidenböck et al., 2000; Seth and Adelman, 2010; Bosch et al., 2012; Kur et al., 2012).

For this reason, mild, moderate and severe categories based only on the number of hemorrhages are insufficient for research. A useful phenotype can be organized around four dimensions: visual consequence, anatomical compartment, barrier status and systemic context. Visual consequence includes acuity, contrast, field symptoms and electrophysiology. Anatomical compartment covers disc, inner retina, outer retina, macula, choroid and vitreous. Barrier status is inferred from fluorescein leakage, OCT fluid or experimentally measured permeability. Systemic context includes oxygen saturation, altitude-illness symptoms, neurological findings, blood pressure and hematocrit. A person with scattered peripheral hemorrhages and normal function is biologically different from a person with a small foveal lesion and photoreceptor disruption, even if a simple count labels the first “more severe”.

### 3.2 Experimental conditions of different exposure patterns

Human studies often pool exposures that should be separated. Acute ascent in a lowlander tests short-term reserve and acclimatization. Chronic residence tests developmental, hemorrhagic and vascular remodeling. Intermittent occupational exposure tests repeated hypoxia–reoxygenation, sleep disruption and work intensity. Chamber studies isolate pressure and oxygen more cleanly, but omit cold, ultraviolet radiation, dehydration, exertion and the behavioral constraints of an expedition. Flight-equivalent exposure is shorter and usually less hypoxic than mountaineering, yet may still reveal pressure-related or perfusion-related ocular responses (Gok et al., 2022; Ma et al., 2022; Ma et al., 2023; Totou et al., 2023; Maria et al., 2024; Westwood et al., 2024; De Abreu et al., 2025; Paillisser et al., 2026).

Lowlanders at altitude commonly show increased retinal venous caliber, tortuosity or changes in choroidal flow (Brinchmann-Hansen et al., 1989; Bosch et al., 2009; MacCormick et al., 2012; Neumann et al., 2016; Westwood et al., 2024; De Abreu et al., 2025). In long-term residents, the phenotype is modified by acclimatization, genetic background, chronic mountain sickness and excessive erythrocytosis. Recent data from highlanders living at very high altitude extend this distinction by testing retinal and choroidal vasoreactivity alongside resting anatomy (Paillisser et al., 2026). A chronically adapted eye may look stable at baseline but have reduced response reserve; conversely, a recent arrival may show conspicuous caliber change while preserving function. Resting measurements and provocation responses answer different questions.

Hematocrit deserves particular attention. A higher red-cell mass can improve arterial oxygen content but increase viscosity and venous pressure. At the retinal level, the balance influences shear stress, transit, capillary perfusion and haemorrhagic risk. Studies in residents with high-altitude polycythemia polycythaemia and in chronic exposure cohorts indicate structural and microcirculatory differences, but cross-sectional designs cannot determine whether a retinal phenotype is protective remodeling, damage or a marker of systemic vascular strain (Ma et al., 2022; Ma et al., 2023). Future work should model hemoglobin and viscosity-related measures as continuous modifiers together with altitude.

### 3.3 Data results under different measurement methods

Fundus photography documents vessel caliber, hemorrhage and disc appearance, while fluorescein angiography reveals leakage that may not be predicted by appearance alone (Willmann et al., 2013). OCT provides layer-resolved thickness, fluid and photoreceptor integrity; OCTA estimates vessel density or flow-related signal but not volumetric flow; functional OCT captures stimulus-related vascular responses. ERG summarizes retinal circuit activity, the photopic negative response emphasizes RGC-related function, and visual evoked potentials include post-retinal conduction. Each method has confounders—including, diurnal variation, axial length, segmentation, adaptation state, electrode placement and temperature, so structure, flow and function should be interpreted together (Janáky et al., 2007; Fischer et al., 2012; Schatz et al., 2013; Li et al., 2025a; Shi et al., 2025; Safarzadeh et al., 2026; Yu et al., 2026a; Yu et al., 2026b).

These measures do not yet define a universal threshold. The appropriate response is not to select a single “best” biomarker but to synchronize repeated measurements and treat their temporal disagreement as part of the signal, moving the field from static grading towards neurovascular phase relationships.

### 3.4 Delayed structural injury and functional recovery

HAR can appear after exposure, sometimes well beyond the point of maximal altitude. Delayed retinal hemorrhages have been described in expedition cohorts and recently in acclimatized lowlanders (Barthelmes et al., 2011; Rana et al., 2025). Several mechanisms could contribute: accumulated endothelial stress, exertion-related venous pressure, reoxygenation-associated oxidative stress change, plasma-volume shifts or simply delayed observation of an asymptomatic lesion. Current evidence does not point to a single mechanism. The practical implication is clearer: a normal examination early in an expedition does not rule out that retinal injury will not emerge later.

Recovery also needs a time axis. Vascular caliber may normalize before or after ERG responses, while focal outer-retinal injury can persist even after the hemorrhage resolves. Longitudinal electrophysiological and structural studies explain why a single post-descent visit is inadequate (Shi et al., 2025). A useful recovery design needs baseline, at least two exposure measurements and more than one post-exposure assessment. It should separate rapid physiological reversibility from slower repair and persistent anatomical loss.

### 3.5 Differential diagnosis of HAR from similar conditions

At altitude, not every retinal hemorrhage is HAR. Hypertension, diabetes, retinal vein occlusion, anemia, thrombocytopenia, Valsalva retinopathy, ocular trauma, inflammatory vasculitis and pre-existing retinal disease can produce overlapping signs. High altitude may precipitate or reveal these conditions, but it does not replace their diagnosis. A unilateral pattern, marked asymmetry, widespread cotton-wool spots, severe visual loss, inflammatory cells, arterial whitening or persistent findings after recovery should widen the differential.

Seek urgent escalation for central visual loss, a dense premacular or vitreous hemorrhage, arterial or venous occlusion, marked disc swelling, severe ocular pain, neurological signs or systemic features of HACE or high-altitude pulmonary edema (HAPE). The eye examination should not delay descent or oxygen in a systemically unwell patient. Conversely, an asymptomatic peripheral hemorrhage in a stable person still deserves documentation, exposure review and follow-up because its prognostic meaning is uncertain.

## 4 The retinal neurovascular unit under hypobaric hypoxia

Evidence boundary. The sections below distinguish direct high-altitude or hypobaric retinal findings from mechanisms extrapolated from other retinal disease, systemic hypoxia or cell models. Direct association is not assumed to establish cell-specific causality.

### 4.1 Dual circulation of the retina and choroid

The retina is not a homogeneous oxygen sensor. Oxygen diffuses into the tissue from two circulations, and the balance varies with retinal depth, light adaptation and systemic oxygenation. Choroidal flow is high and local autoregulation is limited, whereas retinal vessels are more tightly matched to local metabolic demand (Pournaras et al., 2008; Kur et al., 2012; Newman, 2013; Linsenmeier and Zhang, 2017).

Hypoxia perturbs this system through several coupled inputs. Lower arterial oxygen content promotes vasodilation. Hyperventilation lowers carbon dioxide, which can oppose dilation. Sympathetic and endothelial signals change tone, while exercise raises demand and venous pressure. Increased hematocrit raises both oxygen content and viscosity at the same time. Local lactate, nitric oxide (NO), potassium, arachidonic-acid metabolites and adenosine all participate in neurovascular coupling. The observed retinal vessel response is the net result, not a direct readout of one pathway (Pournaras et al., 2008; Bosch et al., 2009; Kur et al., 2012; Newman, 2013; Neumann et al., 2016; Maria et al., 2024). Experimental high-altitude exposure can also increase choroidal thickness and retinal dopamine, further linking perfusion to neural adaptation (Han et al., 2025a).

This helps explain apparently inconsistent human findings. Vessel dilation during acute exposure can be adaptive, yet the same eye may show reduced electrophysiological output if flow no longer matches energy demand. Chronic highlanders may show vascular remodeling or altered reactivity that differs from the acute caliber pattern of lowlanders (De Abreu et al., 2025; Paillisser et al., 2026; Safarzadeh et al., 2026; Yu et al., 2026a; Yu et al., 2026b). An intervention that simply narrows or dilates vessels could therefore be harmful or helpful, depending on the phase. Mechanistic studies need a physiological anchor—oxygen delivery, neural function or barrier integrity—rather than assuming that a direction of caliber change is desirable.

### 4.2 The dual nature of HIF in HAR

Hypoxia-inducible factors (HIFs) drive transcriptional responses to oxygen availability. HIF-1α can support glycolysis, angiogenic signaling, cell survival and vascular adaptation. Sustained or excessive activity can also increase permeability or drive maladaptive angiogenesis, as chronic systemic retinal hypoxia models illustrate (Shortt et al., 2004; Arjamaa and Nikinmaa, 2006). The effect depends on cell type, exposure intensity, duration and crosstalk with HIF-2α and non-HIF pathways. Calling HIF-1α either the “protective target” or the “pathological target” of HAR is therefore too simple.

In endothelium, HIF-dependent signals interact with VEGF, NO and junctional proteins. In Müller glia, hypoxia can induce permeability-promoting mediators such as angiopoietin-like 4 in other retinal models (Xin et al., 2013). In neurons, HIF-related metabolism may preserve ATP initially, but become insufficient as mitochondrial and endoplasmic-reticulum stress accumulate. In salidroside-treated altitude models, HIF-1α/BNIP3-associated mitophagy is linked to protection, whereas Rhodiola extract experiments report benefit alongside downregulation of a HIF-1α/eNOS/NO axis (Li et al., 2025b; Han et al., 2026).

The opposite regulatory vectors are a genuine interpretive tension rather than proof that both directions are therapeutically correct. Different preparations, retinal compartments, doses and exposure phases may move the same HIF network in different directions, but HIF-1α can otherwise become a flexible narrative endpoint. Resolving the paradox requires time-resolved, cell-specific target engagement and causal perturbation, not pathway abundance alone.

### 4.3 Blood–retinal barrier integrity in HAR

The inner BRB is formed by specialized retinal endothelial cells joined by tight and adherens junctions, supported by pericytes, basement membrane, astrocytic processes and Müller endfeet. The outer BRB is formed by retinal pigment epithelium (RPE) junctions. These barriers do more than prevent leakage. They regulate ion, nutrient and metabolite exchange in a tissue that cannot tolerate edema without optical and neuronal consequences (Kaur et al., 2008; Huang, 2020; O'Leary and Campbell, 2023).

Hypoxia can destabilize the inner BRB through oxidative modification, cytoskeletal contraction, junctional phosphorylation, inflammatory mediators and abnormal NO or VEGF signaling. Pericyte–endothelial communication controls capillary stability and local flow; loss of support can increase permeability even before gross vessel disruption (Kaur et al., 2008; Huang, 2020). Müller and astrocytic signals further determine junctional state. This multicellular architecture explains why an endothelial monolayer exposed to low oxygen is useful for dissecting a defined mechanism but cannot reproduce HAR.

Direct experimental studies now connect high-altitude exposure to barrier-related targets. NMN supplementation reduced endothelial ROS-associated Src/PI3K/AKT signaling and protected barrier features in a high-altitude model (Liu et al., 2025). A chemically characterized R. tangutica extract was associated with improved barrier ultrastructure, lower leakage and junctional preservation (Li et al., 2025b). The strength of these studies lies in the combination of barrier endpoints and pathway measurements. Their limitations—pre-treatment, acute exposure, animal models and uncertain human retinal exposure—are equally important.

Vascular leakage also links HAR to hemorrhage. Elevated venous pressure, altered viscosity, endothelial junction failure and mechanical stress during exertion can converge on fragile capillaries. The classic observation that exercise can accompany new hemorrhage is consistent with this interaction, but does not identify a single causal route (McFadden et al., 1981; Brinchmann-Hansen et al., 1989). Modern studies should pair continuous systemic physiology with retinal imaging; the final hemorrhage count alone cannot reveal endothelial biology.

### 4.4 Glial metabolic coupling and lactate homeostasis in HAR

Müller cells span the full retinal thickness. They clear glutamate, buffer potassium, regulate water and supply metabolic substrates. Their endfeet contact vessels and the inner limiting membrane, positioning them between neuronal activity and barrier function. Under hypobaric hypoxia, altered AQP4 and Kir4.1 expression has been associated with Müller-cell edema and retinal injury (Han et al., 2025b). This offers a cellular explanation for why retinal thickening can reflect more than extracellular leakage.

Lactate is another bridge. The retina produces and consumes lactate across cell types; it is both a fuel and a signal. A 2026 altitude study reports that inflammatory stress disrupts retinal lactate homeostasis (Du et al., 2026). The finding is more informative than labelling lactate as waste. If Müller cells, photoreceptors, RGCs and endothelium lose coordinated lactate exchange, blood flow may remain high while neurons become energetically constrained. This possibility connects metabolic mismatch to glial inflammation and offers measurable targets—cell-specific transporters, isotope tracing and extracellular lactate dynamics—rather than another static cytokine panel.

### 4.5 Microglia and the inflammasome in HAR

Microglia survey the retinal environment and respond quickly to damage-associated signals. Acute high-altitude exposure increases retinal microglial activation and migration in experimental work (Li et al., 2025c). Activated microglia can release IL-1β, TNF, ROS and other mediators that weaken endothelial junctions or alter neuronal excitability. Barrier leakage and dying cells then provide additional activating cues. The result is a self-reinforcing inflammatory loop.

P2X7R–NLRP3 signaling has become an important recent target. In a 2026 high-altitude model, P2X7R inhibition protected the retina while suppressing NLRP3-associated pyroptosis (Ge et al., 2026).

### 4.6 Mitochondrial metabolism and antioxidant signaling in HAR

Hypoxia does not halt mitochondrial metabolism uniformly. Reduced oxygen availability reshapes electron transport, membrane potential, substrate choice and ROS generation. Reoxygenation can add a second oxidative challenge. RGCs are vulnerable because axonal transport and electrical activity require sustained ATP, while photoreceptors must support continuous ion flux and outer-segment renewal. Experimental altitude exposure produces RGC structural and functional changes, and systemic hypoxia studies show that glial responses can be substantial even when neuronal loss is limited (Mesentier-Louro et al., 2020; Yang et al., 2024). The difference underlines the need to measure function before assuming cell death.

Time-resolved murine data show that oxidative and apoptotic signals evolve across acute hypobaric exposure, with parallel LIF–JAK–STAT3 activity (Wang et al., 2026a). Transcriptomic work comparing acute and chronic normobaric and hypobaric hypoxia similarly shows that pressure context and duration shape the retinal response (Ebner et al., 2021). A separate miRNA–mRNA network analysis provides hypotheses for regulatory interactions but still needs cell-specific and experimental validation (Su et al., 2021). These studies weaken a common narrative in which ROS rises once, activates inflammation and then causes apoptosis. In reality, redox state, cytokine signaling, substrate availability and organelle turnover change together.

Nrf2–HO-1 is frequently measured because it organizes antioxidant and cytoprotective responses. Hesperidin has direct hypobaric retinal evidence for Nrf2–HO-1-associated protection (Xin et al., 2020), whereas the astaxanthin, curcumin and ginsenoside evidence cited here derives from other retinal injury models (Li et al., 2020; Xie et al., 2021; Li et al., 2024). Such convergence may indicate a tractable stress-response node, but it can also reflect publication habits and shared endpoint selection. Nrf2 protein expression is not equivalent to retinal drug exposure, target dependence or durable functional rescue. Studies should add genetic or pharmacological blockade, dose–response, oxidative-species-specific assays and ERG or visual outcomes.

### 4.7 Organelle stress and quality control in HAR

The endoplasmic reticulum responds to impaired protein folding through PERK, IRE1 and ATF6 branches of the unfolded-protein response. In acute injury, these pathways can restore proteostasis. Persistent activation can contribute to CHOP-associated death. Direct simulated high-altitude animal studies report that pharmacological ER-stress inhibition mitigated retinal damage and that dexamethasone pre-treatment attenuated histomorphological changes in retina and visual cortex (Zheng et al., 2025; Shushanyan et al., 2026). Neither result establishes a clinical retinal indication. They identify stress modules that should be tested against function, timing and systemic effects.

Autophagy and mitophagy present the same interpretive problem. Increased LC3-II can reflect greater autophagosome formation or blocked clearance. A protective conclusion requires flux measurements. Recent salidroside work links HIF-1α/BNIP3-mediated mitophagy with less RGC death and less ferroptotic injury under high-altitude exposure (Han et al., 2026). The proposed relationship is plausible: removal of damaged mitochondria can lower ROS, whereas failed quality control leaves iron-rich, lipid-damaging organelles in place. It remains uncertain whether salidroside directly engages this machinery, changes upstream metabolism or acts through multiple targets.

### 4.8 Regulated cell death in the HAR

Apoptosis, pyroptosis and ferroptosis are distinct experimental programs, but whole-retina marker changes cannot identify the dying cell. Direct altitude studies support RGC structural and functional injury (Yang et al., 2024),and largely whole-retina oxidative and apoptotic signals (Wang et al., 2026a); P2X7R inhibition was associated with reduced NLRP3-related pyroptosis in retinal tissue (Ge et al., 2026); and salidroside-treated animals showed RGC survival with ferroptosis-associated changes (Han et al., 2026). The latter links the phenotype to an RGC outcome, but marker changes still do not prove that every affected RGC died by ferroptosis. One retinal population can undergo inflammatory lysis while another shows apoptotic signaling, and overlapping tissue markers should not be treated as a single cell-specific death program.

Current direct evidence therefore supports involvement of regulated cell-death pathways but remains incomplete for cell-specific causality. The next step has to be causal and spatial. Cell-specific conditional models, lipidomics, iron imaging, membrane-integrity assays and rescue with selective inhibitors should identify the dying cell and the necessary pathway. A drop in TUNEL staining or one protein after a natural compound is not enough to claim that a regulated-death program has been therapeutically controlled.

Independence of evidence is another limitation. A substantial share of recent direct hypobaric retinal studies on RGC injury, AQP4/Kir4.1, dopamine, microglia, ER stress, oxidative and apoptotic dynamics, lactate, P2X7R/NLRP3, barrier signaling and salidroside comes from overlapping investigators and closely affiliated groups (Yang et al., 2024; Han et al., 2025a; Han et al., 2025b; Li et al., 2025c; Liu et al., 2025; Zheng et al., 2025; Du et al., 2026; Ge et al., 2026; Wang et al., 2026a; Han et al., 2026). This coherent research program is valuable, but it should not be counted as multiple independent confirmations. Multicenter replication with shared chamber metadata, masked analysis and external validation is needed before these mechanisms are treated as settled HAR biology (Figure 2).

**FIGURE 2:**
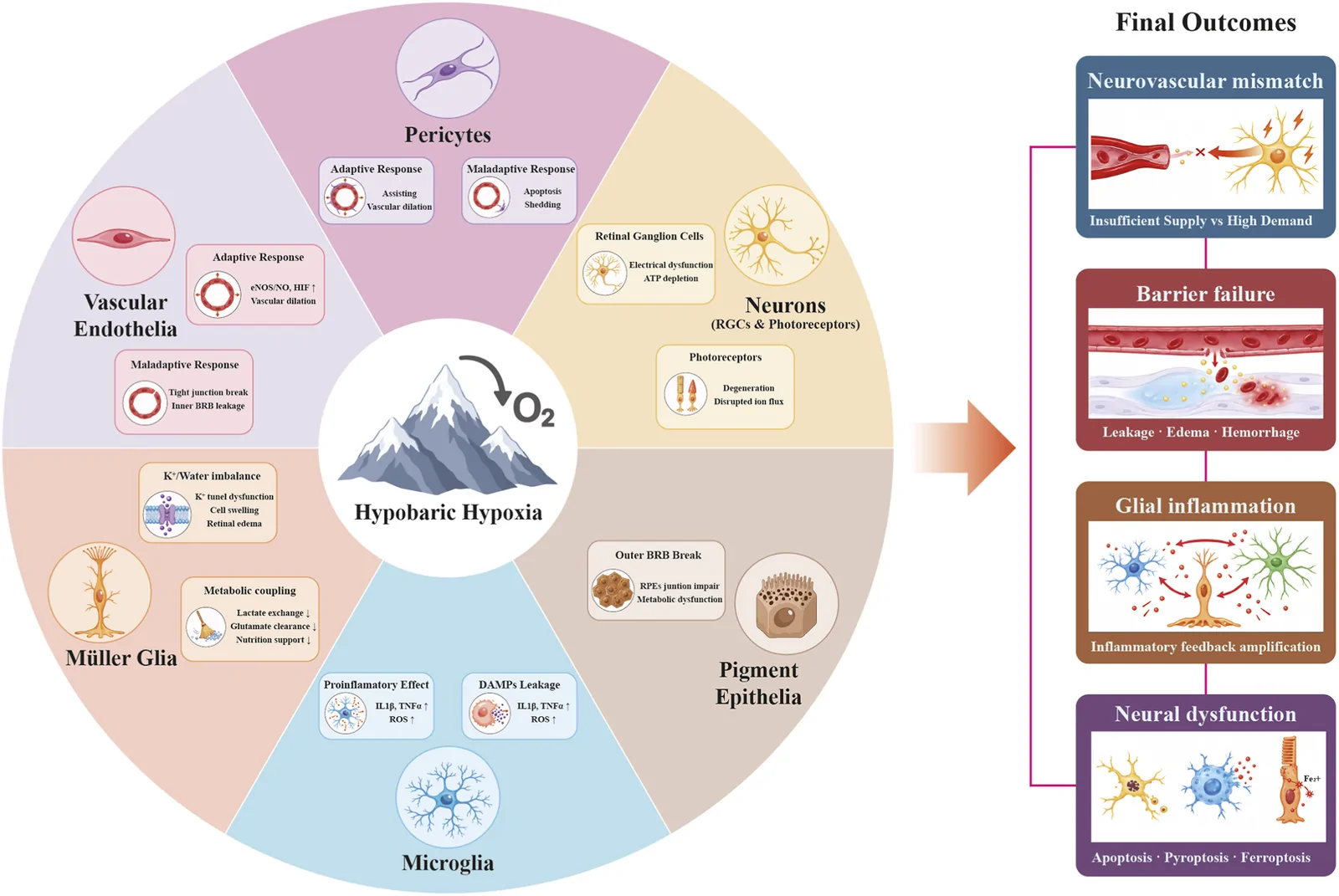
Cell-specific responses of the retinal neurovascular unit to hypobaric hypoxia and their integrated pathological outcomes. Hypobaric hypoxia drives cell-specific adaptive and maladaptive responses across the retinal neurovascular unit. Endothelium: transient HIF and eNOS/NO activation plus pericyte control of capillary tone supports vasodilation; sustained hypoxia causes tight-junction disruption and pericyte loss, driving inner BRB leakage. RGCs/photoreceptors: oxygen/substrate deficit relative to demand leads to ATP depletion, electrical dysfunction, disturbed ion flux, and degeneration. RPE: junctional and metabolic failure compromises the outer BRB. Microglia: DAMPs trigger IL-1β, TNF-α, and ROS, closing an inflammatory loop. Müller glia: disrupted K^+^/water homeostasis, lactate exchange, glutamate clearance, and metabolic support promote cellular swelling and edema. These converge on neurovascular mismatch, barrier failure (leakage, edema, hemorrhage), glial amplification, and neural death (apoptosis, pyroptosis, ferroptosis). Pathways are interconnected, not linear. ATP, adenosine triphosphate; BRB, blood–retinal barrier; DAMPs, damage-associated molecular patterns; eNOS, endothelial nitric oxide synthase; HIF, hypoxia-inducible factor; IL-1β, interleukin-1β; NO, nitric oxide; RGCs, retinal ganglion cells; ROS, reactive oxygen species; RPE, retinal pigment epithelium; TNF-α, tumor necrosis factor-α.

## 5 Modern clinical research

### 5.1 Acute altitude illness and the retinal examination

There is no HAR-specific drug regimen supported by randomized human trials. The most defensible intervention is exposure control. A person with new visual symptoms, severe headache, vomiting, ataxia, altered mental status, dyspnea at rest or falling oxygen saturation should stop ascent. Descent and supplemental oxygen are central measures for significant altitude illness; a portable hyperbaric chamber is a bridge when descent is temporarily impossible, not a substitute for evacuation (Luks et al., 2017; Luks et al., 2024; McLaughlin et al., 2026). The retinal examination should proceed in parallel only when it does not delay systemic care.

Mild asymptomatic retinal findings require a more measured response. The exposure history should include maximum altitude, ascent rate, sleeping altitude, exertion, hydration, medications, previous altitude illness and pre-existing ocular or hematologic disease. Visual acuity, pupils, fields where feasible, blood pressure, oxygen saturation and neurological status establish context. Fundus photography documents the lesion; OCT determines macular, disc and outer-retinal involvement. OCTA and ERG add research value and may aid follow-up, but normal results do not erase a documented hemorrhage.

### 5.2 Limits of extrapolating altitude-medicine drugs to HAR

Acetazolamide reduces risk and treats symptoms of AMS by promoting ventilatory acclimatization. Dexamethasone is used in prevention or treatment of selected severe altitude-illness settings, including HACE, under established guidance (Luks et al., 2017; Luks et al., 2024). Their clinical evidence does not use HAR endpoints. A 2026 rat study of dexamethasone pre-treatment is mechanistically relevant, but cannot convert systemic guideline use into an HAR-specific recommendation (Shushanyan et al., 2026). The manuscript therefore avoids statements such as “acetazolamide treats retinal edema” or “dexamethasone prevents HAR”.

The same boundary applies to nifedipine, phosphodiesterase inhibitors and other agents used for HAPE prevention or rescue. They can change systemic hemodynamics and oxygenation, which may secondarily affect the retina, but direct retinal benefit has not been established. A 2025 joint recommendation for unacclimatized rescuers reviews oxygen and pharmacological prophylaxis at very high altitude; its operational context should not be mistaken for evidence in an ophthalmic population (McLaughlin et al., 2026). This evidentiary boundary does not preclude clinical judgment in remote emergencies. When evacuation is delayed or impossible, established rescue protocols for AMS, HACE or HAPE may be used empirically according to wilderness-medicine guidance; the point is that such use should not be presented as proof of HAR-specific retinal efficacy.

### 5.3 Ocular diagnosis–directed management of HAR

Once exposure has been addressed, management follows the ocular diagnosis. A small peripheral hemorrhage with preserved function may be observed with documented recovery. Dense premacular or vitreous hemorrhage, macular edema, retinal vein or artery occlusion, retinal tear or detachment and ischemic optic neuropathy require specialist assessment. Anti-VEGF therapy, laser, vitrectomy, displacement procedures or other interventions should be considered only under their complication-specific indications. HAR itself is not a blanket indication for intravitreal therapy.

This distinction is clinically useful and rhetorically important. Case reports can show what occurred after an intervention, but cannot establish comparative efficacy or prophylaxis. Recent cases extend the recognized phenotype to bilateral macular hemorrhage with brain edema, isolated ellipsoid-zone loss and prolonged “blue-spot” symptoms (Smith et al., 2025; Bailey et al., 2026; Zając et al., 2026). They support careful imaging and follow-up. They do not define a standard procedure for all HAR.

### 5.4 Follow-up and return to altitude after HAR

Follow-up timing should depend on the lesion and symptoms. Central hemorrhage, outer-retinal disruption, disc edema or functional deficit merit repeated OCT and visual assessment until stable. Where electrophysiology is abnormal, acuity alone is insufficient to document recovery. Persistent or asymmetric findings should prompt reconsideration of the diagnosis.

Evidence for return to altitude after HAR is sparse. A cautious decision considers residual structural damage, previous severe altitude illness, required rate of ascent, ability to descend, occupational constraints and underlying vascular risk. Gradual ascent and early symptom recognition are reasonable, but no natural product or ophthalmic drug can currently be recommended to make re-ascent safe. Prospective registries are needed before confident return-to-altitude rules can be written (Box 1; Tables 1, 2; Figure 3).

BOX 1 **Field-to-clinic approach for suspected HAR.**

1. Check visual symptoms and red flags. Ask about visual change and severe altitude illness. Neurological signs, dyspnea at rest or rapid deterioration require immediate escalation.
2. Stop ascent. Do not continue gaining altitude while significant visual or systemic symptoms are evolving.
3. Treat hypoxia and reduce exposure when clinically indicated. Give oxygen and descend. If descent is temporarily impossible, use established rescue measures without delaying evacuation.
4. Perform urgent systemic assessment. Assess mental status, gait, respiratory distress, oxygen saturation and HACE/HAPE features.
5. Document and localize the ocular lesion. Record acuity, pupils, fundus photographs and OCT/OCTA where feasible; distinguish peripheral hemorrhage from macular, disc, vascular-occlusive, vitreous or outer-retinal involvement and review competing causes.
6. Add functional testing when feasible. ERG or another validated functional measure can support research and follow-up but must not delay emergency care.
7. Use complication-specific care and plan recovery assessment. HAR alone is not an indication for anti-VEGF injection, laser or surgery; repeat examination after descent when symptoms, macular changes or disc involvement persist.

**TABLE 1:** Clinical features and recommended assessment.

Assessment area	Clinical features	Recommended assessment	Considerations for interpretation
Retinal vasculature	Venous dilation, tortuosity, altered capillary signal	Standardized fundus photography; OCTA; vessel analysis	Visible area or caliber is not identical to oxygen delivery or volumetric flow
Choroidal circulation	Thickness or layer-specific flow change	Enhanced-depth OCT; functional OCT; flowmetry where available	Choroidal thickness is influenced by diurnal, axial-length and hydration effects
Neuroretinal function	Reduced ERG component, delayed response, contrast or field symptom	Full-field or pattern ERG; PhNR; visual field; contrast sensitivity	Normal acuity does not exclude inner-retinal or localized outer-retinal dysfunction
Blood–retinal barrier	Leakage, intraretinal fluid, disc edema	Fluorescein angiography; OCT; experimental permeability assays	Thickening alone cannot distinguish vascular filling, cell swelling and extracellular fluid
Retinal structure	Hemorrhage, ellipsoid-zone loss, RGC or RNFL change	Colour/wide-field imaging; OCT; adaptive optics in research	A small foveal lesion may be more disabling than numerous peripheral lesions
Systemic altitude illness	AMS/HACE/HAPE symptoms, hypoxemia, polycythaemia	SpO_2_, symptom and neurological assessment, blood pressure, haemoglobin/hematocrit	Retinal findings complement but do not diagnose or exclude cerebral or pulmonary altitude illness

**TABLE 2:** Core outcomes for a translational HAR study.

Research domain	Preclinical measure	Clinical correlates	Key translational questions
Exposure and dosing	Chamber pressure/gases; plasma and retinal drug/metabolite concentrations	Altitude exposure log; SpO_2_; dosing; plasma pharmacokinetics when ethically feasible	Was the hypoxic and pharmacological exposure achieved?
Vascular integrity	Vessel caliber, OCTA, flowmetry, permeability	Standardized fundus photography; OCTA; fluorescein angiography when indicated	Is compensation preserved and is the BRB intact?
Neural function	ERG/PhNR, visual behaviour, RGC/photoreceptor survival	ERG/PhNR; visual acuity; contrast sensitivity, visual fields; symptoms	Does vascular change translate into neural benefit?
Metabolic function	Lactate tracing; mitochondrial respiration; NAD^+^/redox assays	Blood markers plus non-invasive retinal physiology; exploratory metabolomics	Is energy mismatch corrected at an achievable exposure?
Inflammation and cell death	Cell-specific microglial state; gasdermin cleavage; lipid peroxidation; rescue experiments	Exploratory fluid biomarkers; imaging; functional recovery	Is the proposed mechanism necessary and linked to outcome?
Recovery	Serial measures after reoxygenation	Repeated post-descent OCT/OCTA/ERG	Is benefit durable, and are vascular and neural recovery synchronized?
Safety	Hemodynamics; hematology; organ and ocular toxicity	Adverse events; drug interactions; blood pressure; laboratory tests	Can the intervention move to preventive human study?

**FIGURE 3:**
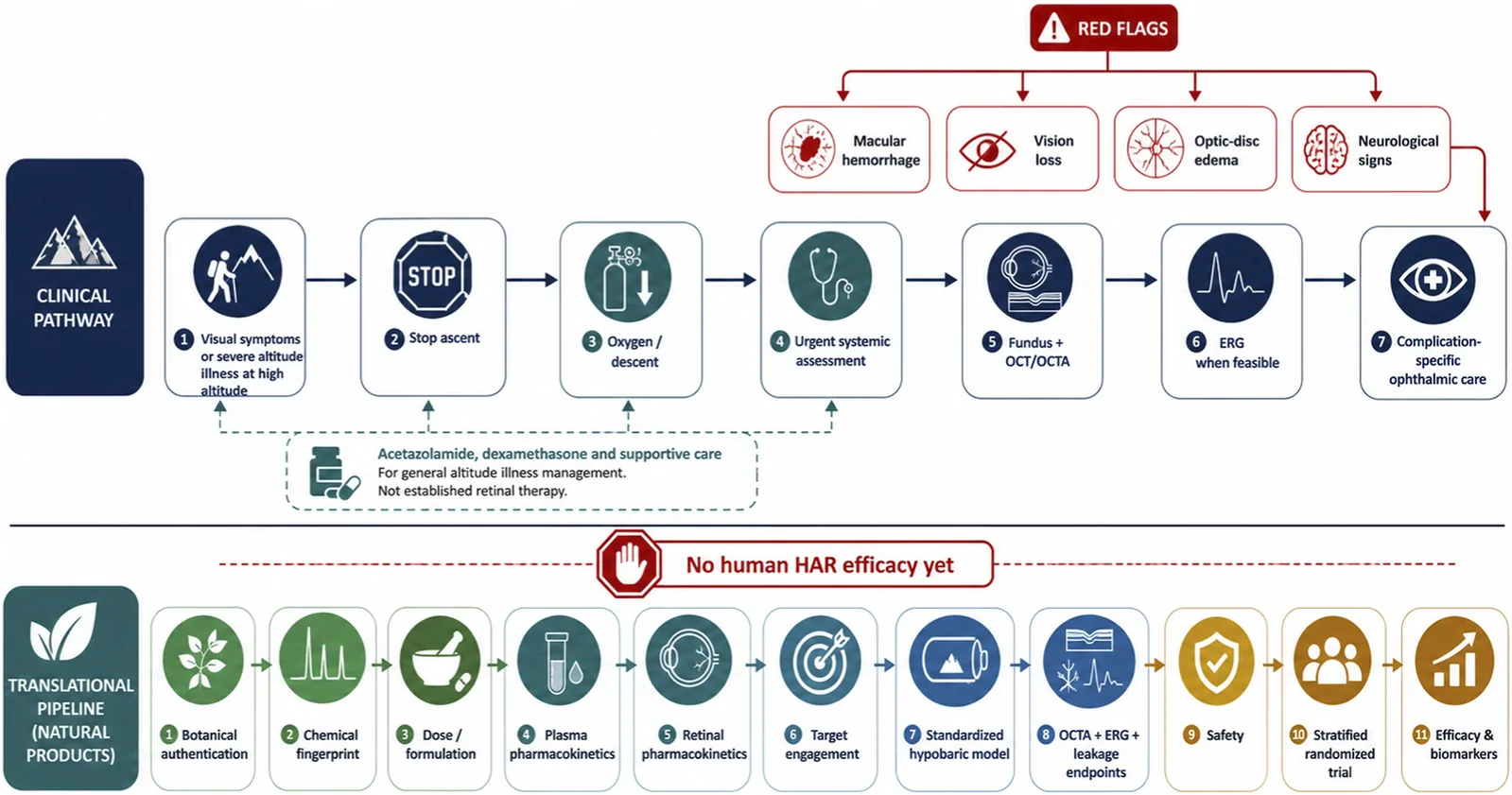
Clinical pathway and translational gate. The clinical track prioritizes exposure control, systemic assessment and complication-specific ophthalmic care. Acetazolamide and dexamethasone belong to general altitude-illness management and are not established HAR-specific retinal treatments. The natural-product track requires authentication, chemistry, pharmacokinetics, target engagement, standardized models, functional endpoints and safety before human efficacy can be tested. The numbered clinical steps correspond directly to Box 1; emergency assessment and exposure correction may proceed in parallel when required.

## 6 Natural compounds and extracts

### 6.1 Why this field needs an evidence hierarchy

Natural products are attractive in altitude research because many affect redox state, inflammation, vascular signaling and mitochondrial metabolism. These broad actions are also a liability. A compound can lower several stress markers without reaching the retina at an achievable exposure, and an extract can appear reproducibly active while its composition varies between batches. The question is not whether a preparation is “natural” or historically used. It is whether a chemically defined intervention changes a disease-relevant retinal outcome through a testable mechanism at a plausible dose.

Four evidence tiers are useful. Tier 1 is a human HAR trial with retinal endpoints; no candidate meets it. Tier 2 is a direct hypobaric or high-altitude retinal model with structural or functional outcomes. Salidroside, a characterized R. tangutica extract, resveratrol and hesperidin meet this preclinical tier (Xin et al., 2017; Xin et al., 2020; Li et al., 2025b; Han et al., 2026). Tier 3 is another retinal hypoxia, ischemia, diabetic or excitotoxic model that supports a mechanism but requires extrapolation. Tier 4 comprises isolated pathway, docking or network-pharmacology results without adequate retinal validation. Tier 4 can generate hypotheses but should not carry efficacy language.

The distinction between compounds and extracts must remain explicit. Salidroside is a defined phenylpropanoid glycoside. A Rhodiola extract contains multiple constituents whose amounts depend on species, plant part, geography and processing. Evidence for salidroside does not validate any commercial Rhodiola product. Conversely, an extract study cannot attribute its effect to salidroside without fractionation, exposure data or causal testing.

### 6.2 Salidroside

A recent direct Tier 2 preclinical study examines salidroside in acute high-altitude exposure. The reported protection includes retinal structure and function, RGC survival and changes consistent with HIF-1α/BNIP3-mediated mitophagy and reduced ferroptotic stress (Han et al., 2026). This work is important because it moves beyond a generic “antioxidant” label and connects an intervention to organelle quality control and a regulated-death program. It also raises a harder question: whether the observed HIF and mitophagy changes are the necessary target, a compensatory marker or one component of a multi-target response.

Supporting retinal studies broaden the possible cell targets. In hypoxic human retinal microvascular endothelial cells, salidroside has been linked to a miR-138/ROBO4 axis (Shi et al., 2021). Oxidative-injury studies in RPE and retinal endothelium report less stress and apoptosis (Shi et al., 2015; Yin et al., 2018). Diabetic retinal models suggest effects on Müller-cell inflammation, RGC apoptosis and NLRP3-associated pyroptosis (Yao et al., 2022; Li et al., 2023; Zhang et al., 2024). A 2026 study links salidroside to endothelial glycolysis and hypoxia-induced ocular angiogenesis through PFKFB3 acetylation (Li et al., 2026). These studies show mechanistic breadth, not a single proven chain in HAR.

### 6.3 Rhodiola tangutica extract

A 2025 direct Tier 2 preclinical study of R. tangutica extract in hypoxia-induced retinal injury reported improved BRB-related outcomes and downregulation of a HIF-1α/eNOS/NO pathway (Li et al., 2025b). Its value lies in measuring barrier integrity as well as systemic altitude responses. Chemical characterization also makes the experiment more interpretable than work with an unnamed crude decoction.

The extract nevertheless presents a different regulatory problem from salidroside. Botanical identity, voucher specimen, plant part, extraction solvent, drug-to-extract ratio, chromatographic fingerprint, quantified markers and contaminant testing must travel with every efficacy claim. If the extract is standardized to salidroside alone, other abundant or active constituents can still vary. Batch equivalence should be tested analytically and, where possible, with a small set of biological quality attributes.

Mechanistically, reported downregulation of HIF-1α/eNOS/NO should not be generalized into “HIF inhibition is protective”. NO regulates retinal tone and can be adaptive. Excessive or spatially inappropriate NO, interaction with ROS and junctional effects may be harmful. The useful next experiment would combine retinal exposure, endothelial target engagement, vessel reactivity, leakage and ERG across exposure phases. That design could determine whether the extract preserves useful vasodilation while preventing barrier failure. Direct comparison of salidroside and the extract within the same model, with cell-specific timing and causal HIF perturbation, is needed before either regulatory direction is labeled therapeutic.

### 6.4 Resveratrol

In a direct Tier 2 rat hypobaric-hypoxia study, resveratrol reduced retinal injury-related markers and improved histological features (Xin et al., 2017). In retinal ischemia–reperfusion, it delays RGC loss, reduces gliosis-associated inflammation and suppresses an NLRP3/GSDMD/caspase-1/IL-1β axis (Luo et al., 2018; Xie et al., 2023). Other models suggest effects on endoplasmic-reticulum stress, OPA1-associated mitochondrial dynamics and RPE bioenergetics (Li et al., 2012; Sheu et al., 2013; Pang et al., 2020). These data make resveratrol a useful mechanistic probe across vascular, glial and mitochondrial compartments.

They do not solve its pharmacology. Resveratrol undergoes rapid metabolism, and the active species in plasma or retina may not be the parent compound. Intraperitoneal or high-dose animal protocols can create exposures that oral human supplementation does not reproduce. Formulations designed to increase ocular delivery may alter distribution and safety as much as efficacy. Any HAR program should measure parent compound and major metabolites in plasma and retinal tissue, report parent and major-metabolite exposure, with unbound concentrations where technically feasible, and compare them with the concentration required for target modulation.

The molecule’s popularity creates an additional bias. A large literature increases the number of positive mechanisms available for citation, but not the certainty of an HAR indication. The direct altitude study should remain the anchor; ischemia and RPE studies define questions for target engagement and formulation, not evidence that climbers should take resveratrol.

### 6.5 Hesperidin

Hesperidin also has direct Tier 2 hypobaric retinal evidence in rats, where it was associated with Nrf2–HO-1 activation, lower oxidative and apoptotic markers and improved retinal injury measures (Xin et al., 2020). Diabetic and NMDA injury models support redox and neuroprotective effects, including suppression of excessive calpain activation (Shi et al., 2012; Maekawa et al., 2017). A 2026 nanoparticle study in oxygen-induced retinopathy adds immune-microenvironment and delivery questions (Wang et al., 2026b).

The major uncertainty is exposure. Hesperidin is metabolized by the gut microbiota and host enzymes; circulating metabolites may differ substantially from the parent glycoside. Retinal concentrations are rarely aligned with the dose and molecular readout. Nanoformulation can improve delivery but creates a new product whose carrier, size, release, immune interaction and ocular distribution require independent evaluation. Results from pathological angiogenesis should also not be merged with acute HAR, where suppressing all vascular adaptation could be counterproductive.

The most informative next study would compare hesperidin, its principal circulating metabolites and a defined formulation in the same hypobaric model, with plasma/retinal pharmacokinetics and synchronized OCTA–ERG endpoints. That design would separate a chemically attractive hypothesis from an achievable intervention.

### 6.6 Other natural candidates

Baicalein, baicalin and wogonin have protective evidence in cerebral and retinal ischemia models (Pan et al., 2021). Mitochondria-targeted quercetin formulations reduce injury in retinal ischemia–reperfusion and illustrate how delivery can be coupled to a redox mechanism (Zhao et al., 2023). Curcumin, ginsenoside Rg3 and astaxanthin affect Nrf2-related, inflammatory or endoplasmic-reticulum pathways in diabetic or pressure-related retinal injury (Li et al., 2020; Xie et al., 2021; Li et al., 2024). These compounds are reasonable candidates for comparative screening because they touch processes now implicated directly in HAR. They are not members of the same evidence tier as salidroside, Rhodiola extract, resveratrol or hesperidin.

The wider ocular nutraceutical literature reinforces this caution. Reviews of plant-derived retinal interventions emphasize BRB delivery, while a 2026 synthesis of food-derived bioactives concludes that much of the polyphenol evidence remains preclinical and heterogeneous (Ren et al., 2024; Paracini et al., 2026). Lutein and zeaxanthin have human evidence in macular health, but that evidence concerns a different disease context and cannot be imported into HAR. A compound’s safety as a food constituent also does not establish safety at pharmacological doses, during dehydration, with altitude medications or in people with polycythaemia.

Comparative studies should not begin with twenty compounds and one oxidative marker. A useful screen would select a small set based on distinct pharmacology, test achievable systemic and, where feasible, retinal exposures, include a positive control tied to the model, and require improvement in at least one functional and one structural or barrier endpoint. Candidates that pass would proceed to cell-specific target engagement and reoxygenation studies (Box 2; Table 3; Figure 4).

BOX 2 **Stage-appropriate reporting standards for natural-product HAR studies.**

Identity: species, authority, plant part, voucher specimen and geographical source for botanicals; purity and stereochemistry for isolated compounds.

Manufacture: extraction solvent, temperature, duration, drug-to-extract ratio, excipients and batch number.

Chemistry: isolated compounds require identity and purity; extracts require a reproducible chromatographic fingerprint and quantified marker panel scaled to the study stage, with analytical performance and contaminant reporting.

Exposure: exploratory studies should justify dose and report plasma exposure where feasible; confirmatory studies should quantify parent and major metabolites in plasma and total retina with contamination controls. Unbound retinal concentrations are an aspirational gold standard where technically valid, not a universal minimum.

Model: barometric pressure, oxygen and carbon dioxide, ascent profile, exposure duration, reoxygenation, temperature, activity, sex, age and randomization/blinding.

Outcomes: at least one functional endpoint (ERG, PhNR or validated visual behaviour) plus OCT/OCTA, leakage or histological endpoints.

Mechanism: exploratory studies should pre-specify a plausible retinal target and localize the response; confirmatory studies should demonstrate dose–response, target engagement and, where feasible, causal perturbation in the relevant cell.

Safety: systemic hemodynamics, hematology, liver/kidney measures, ocular toxicity and interaction with standard altitude-illness treatment.

Claim: name the evidence tier and state explicitly whether human HAR efficacy has been tested.

**TABLE 3:** Evidence map for natural compounds and extracts.

Intervention	Direct evidence in HAR/high-altitude retinal models	Supporting evidence from other retinal models	Key translational gap	Current conclusion
Salidroside	Acute high-altitude mouse model with structural, functional, mitophagy and ferroptosis-related outcomes (58)	Hypoxic retinal endothelium, oxidative RPE/endothelial injury, diabetic retinal inflammation and pyroptosis, hypoxic angiogenesis (62–67, 80)	Parent/metabolite retinal exposure; causal target dependence; chronic and recovery models	Direct preclinical lead; no human HAR efficacy
Chemically characterized R. tangutica extract	Hypoxia-induced retinal injury model with BRB and hemodynamic endpoints (59)	Broader rhodiola altitude literature is not equivalent to retinal evidence	Batch comparability, marker panel, active constituents, retinal PK	Extract-level preclinical lead; cannot be reduced to salidroside alone
Resveratrol	Rat hypobaric-hypoxia retinal study (60)	Retinal ischemia–reperfusion, RGC, glial, ER-stress and mitochondrial models (68–72)	Rapid metabolism, formulation dependence, active species, achievable ocular exposure	Mechanistically coherent but pharmacokinetically unresolved
Hesperidin	Rat hypobaric-hypoxia retinal study (61)	Diabetic, excitotoxic and oxygen-induced retinopathy models (73, 74, 81)	Microbial/host metabolites, retinal exposure, effect on adaptive vascular responses	Direct preclinical signal; human bridge absent
Baicalein/baicalin, quercetin, curcumin, ginsenoside Rg3, astaxanthin	None identified in direct HAR models	Retinal ischemia, diabetes, excitotoxicity or pressure-related injury (75–79)	Disease-model mismatch and uncertain ocular PK	Repurposing horizon only; do not claim HAR efficacy

**FIGURE 4:**
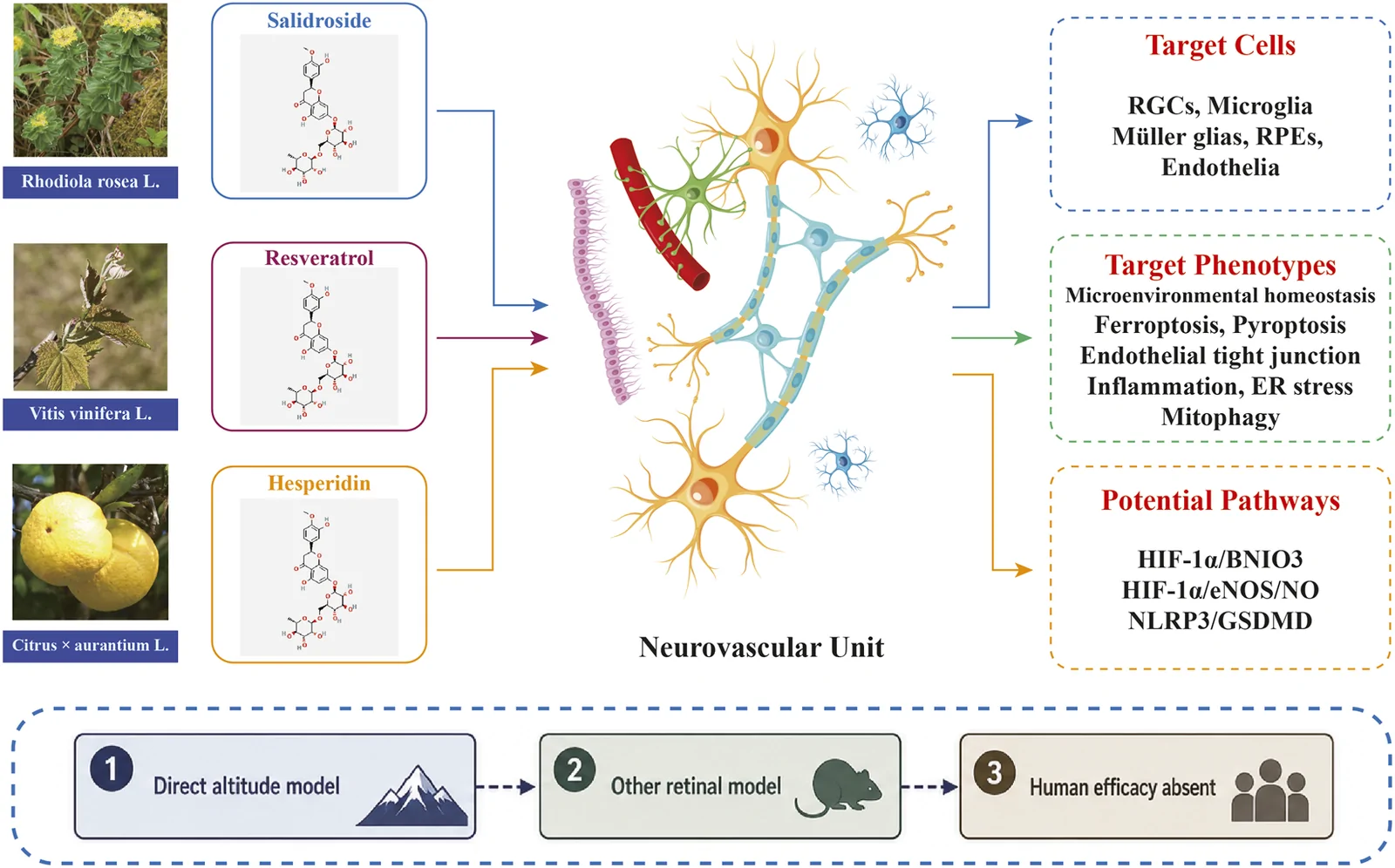
Natural-product candidates, retinal targets, and evidence boundaries in high-altitude retinopathy. Left: botanical sources and candidate compounds-salidroside (Rhodiola rosea L.), resveratrol (Vitis vinifera L.), hesperidin (Citrus × aurantium L.). Center: retinal neurovascular unit (neurons, Müller glia, microglia, RPE, endothelium, mural cells). Reported/proposed effects across RGCs, microglia, Müller glia, RPE, and endothelium: preserved tight junctions, reduced inflammation/ER stress, tuned mitophagy, limited ferroptosis/pyroptosis. Pathways: HIF-1α/BNIP3 mitophagy, HIF-1α/eNOS/NO, NLRP3/GSDMD pyroptosis. Bottom: evidence hierarchy. Direct data from experimental high-altitude/hypobaric retinal models, supplemented by other retinal disease models; human HAR efficacy unproven. Aggregated associations across the three compounds, not implying each acts on every cell/pathway. BNIP3, BCL2-interacting protein 3; eNOS, endothelial nitric oxide synthase; ER, endoplasmic reticulum; GSDMD, gasdermin D; HIF-1α, hypoxia-inducible factor 1α; NLRP3, NOD-like receptor family pyrin domain-containing 3; NO, nitric oxide; RGCs, retinal ganglion cells; RPE, retinal pigment epithelium.

## 7 Current gaps in research

### 7.1 Standardize what “high altitude” means in an experiment

Hypobaric-hypoxia studies often report an equivalent altitude but omit the pressure trajectory, oxygen fraction, carbon dioxide, humidity or temperature. Normobaric hypoxia is then discussed as though it were interchangeable. Transcriptomic comparisons indicate that normobaric, and hypobaric exposures and acute versus chronic duration can produce different retinal responses (Ebner et al., 2021). A minimum model description should therefore include absolute pressure, inspired gases, ascent and descent rates, duration, reoxygenation and environmental conditions. If altitude is simulated by pressure, the calibration and chamber variability should be reported.

Composite stress should be introduced deliberately. Cold, ultraviolet exposure, exercise, dehydration, sleep disruption and circadian timing are genuine features of high-altitude work and travel. Adding all at once creates an uninterpretable model. A better sequence is: define the hypobaric effect; add one modifier with a factorial design; then test a field-relevant composite. This approach can identify whether a candidate protects against low oxygen itself or against a secondary stressor. A field-relevant composite is therefore an aspirational validation model rather than a universal minimum; the immediate priority is transparent reporting of the variables controlled, measured and left uncontrolled.

Acute and chronic questions also need separate models. A prophylactic compound for rapid ascent should be tested before and during acute exposure, including early recovery. A candidate for chronic residents should be studied after remodeling has developed, not only as pre-treatment. Repeated hypoxia–reoxygenation may better represent flight crews or rotational workers. The same molecular endpoint can mean different things in these settings.

### 7.2 Use the neurovascular unit as the experimental unit

Isolated endothelial, RPE or 661 W cells are valuable for perturbation, but they lack flow, pericytes, immune cells and the dual retinal–choroidal circulation. Retinal explants preserve architecture but lose systemic inputs. Rodent models add circulation and behaviour but differ from the human macula. No model is complete; a linked model stack is more credible than claiming that one is.

A practical stack begins with a relevant cell system for target dependence, moves to a co-culture or organotypic model for cell communication, then tests a standardized hypobaric animal model with OCT/OCTA and ERG. Spatial transcriptomics or single-cell sequencing can identify cell states, but the final mechanistic claim should return to protein localization, metabolite flux and causal manipulation. Omics is a map, not target validation.

Pericytes and Müller glia deserve more attention in this stack. They regulate flow, junctions, water, potassium and lactate yet are often absent from screening. Microglial state should be measured beyond an M1/M2 label, which compresses a continuum of injury, repair and metabolic phenotypes. Cell-resolved temporal sampling during exposure and reoxygenation is likely to reveal why vascular and neural recovery diverge.

### 7.3 Make pharmacokinetics part of the mechanism

Retinal pharmacokinetics should be built in early, not left as an optional late step. Plasma exposure alone cannot show retinal target availability: the BRB restricts entry and can change with injury. For exploratory work, dose rationale, systemic exposure, and carefully collected total-retina concentrations are a practical first step. Confirmatory studies should quantify parent drug and major metabolites in plasma and retina across ascent, sustained hypoxia, and recovery, with perfusion and checks for contamination from choroid, vitreous, or residual blood.

Measuring unbound drug in rodent retina is difficult. Treat it as an ideal standard, not a requirement for every publishable study. Microdialysis, homogenate equilibrium dialysis, and model-based estimates all carry assumptions. Clear reporting of total tissue exposure and method limits lets early-stage labs contribute while showing what evidence is still needed for translational claims.

For extracts, a marker may establish batch identity without being the active constituent. Exposure–response analysis should track several plausible constituents and metabolites against a combined pharmacodynamic readout. If no constituent reaches a plausible retinal concentration, effects may be systemic and should be described as such.

Formulation is part of the intervention. Nanoparticles, liposomes, or other delivery systems can change distribution, half-life, immune interaction, and toxicity. Compare the formulated product with free compound at matched exposure, not matched dose. Delivery gains matter only if safety and retinal target engagement improve.

### 7.4 Choose endpoints that can cross from animals to humans

Histology is useful but terminal. OCT, OCTA and ERG can be repeated in animals and humans, making them stronger translational endpoints. A core preclinical set should include fundus imaging, layer-specific OCT, vascular or leakage measurement, ERG/PhNR and a functional visual assay. Molecular endpoints should explain the phenotype, not replace it.

Human cohorts should measure the same domains at baseline, during exposure and recovery. Oxygen saturation, end-tidal or arterial carbon dioxide where feasible, blood pressure, haemoglobin/hematocrit, activity and symptom scores are necessary covariates. A study that measures OCTA without carbon dioxide or hematocrit can misattribute a systemic vascular effect to local retinal biology.

A composite “HAR score” should not be invented by adding unrelated measurements with equal weights. Prediction models require pre-specified outcomes, adequate sample size, internal validation and ideally external replication. A more transparent approach is to define state transitions: preserved function with vascular change; functional decline without leakage; barrier failure; structural injury; incomplete recovery. These states can support trial stratification without pretending that one number captures the disease.

### 7.5 A feasible first clinical trial

The first human natural-product trial should not enrol all travellers and use headache as the endpoint. It should begin only after retinal exposure and safety are credible. A feasible design would recruit healthy lowlanders with a standardized ascent profile, exclude major retinal and hematologic disease, and randomize a chemically defined product against placebo. Baseline, early-altitude, peak-exposure and recovery visits would combine fundus photography, OCT/OCTA and a portable functional measure. Systemic altitude symptoms and oxygenation would be secondary or contextual endpoints, not substitutes for retinal outcomes.

The primary outcome should be a pre-specified retinal state or continuous measure with known repeatability, adjusted for baseline, carbon dioxide, hematocrit and image quality. Power calculations should use that endpoint; broad mechanistic panels are secondary. A nested pharmacokinetic–pharmacodynamic analysis can test whether exposure predicts vascular–functional coupling. Severe symptoms would trigger standard care and withdrawal from further ascent regardless of study assignment.

Clinical translation in chronic residents is a different program. It requires stable phenotyping, attention to excessive erythrocytosis and long-term safety. An intervention that blunts acute vascular adaptation in a lowlander could be inappropriate in a chronic highlander. Stratification is not a decorative final step; it follows directly from the biology.

### 7.6 Pragmatic hurdles in implementation

Claim strength should match evidence tier, not exclude early work. Discovery studies confirm material identity, model conditions, and at least one functional plus one structural/barrier outcome; mechanistic studies add exposure–response, retinal localization, and target engagement; translational studies add retinal pharmacokinetics, safety, formulation controls, and independent replication. Early studies remain useful if claims stay within their tier.

Lack of HAR-specific retinal trials does not invalidate clinical judgment or altitude-emergency protocols. When evacuation is delayed in austere settings, rescue, oxygen, descent, teleconsultation, and empiric treatment of suspected AMS, HACE, or HAPE may proceed by risk. Document this as emergency care, not evidence for treating retinal injury.

A hypobaric chamber can support mechanistic work if it reports temperature, humidity, gases, ascent rate, duration, and reoxygenation. Add cold, ultraviolet exposure, dehydration, and exertion in staged, hypothesis-driven designs. Full field simulation is an ideal validation model, not a minimum requirement (Box 3).

BOX 3 **Claims supported and not supported by current evidence.**

**Supported**

High altitude alters retinal and choroidal vascular, structural and functional measurements in humans.

Vascular and neural responses can be temporally uncoupled during hypoxia and recovery.

Experimental HAR involves BRB, glial, metabolic, organelle-stress and regulated cell-death mechanisms.

Salidroside, characterized R. tangutica extract, resveratrol and hesperidin have direct preclinical high-altitude retinal evidence.

Descent, oxygen and management of concomitant altitude illness are the clinically defensible priorities in significant illness.

**Not supported**

A single OCTA, disc or hemorrhage measure diagnoses or excludes HACE.

Acetazolamide, dexamethasone or anti-VEGF therapy is an established universal HAR treatment.

Evidence from diabetic retinopathy, ischemia–reperfusion or oxygen-induced retinopathy proves efficacy in HAR.

A salidroside study validates an unspecified Rhodiola supplement, or an extract study proves salidroside is the active constituent.

Any natural product has demonstrated preventive or therapeutic efficacy for HAR in humans.

## 8 Future priorities

### 8.1 Establish natural history before treatment effect

The field needs larger longitudinal cohorts across recent arrivals, chronic residents, rotational workers, flight personnel and people with previous altitude illness. Repeated imaging and function should begin before ascent and continue after descent. Harmonized protocols would allow individual trajectories to be compared across sites. Without natural history and measurement repeatability, small changes after a compound cannot be separated from acclimatization or regression to the mean.

### 8.2 Resolve susceptibility

Not everyone exposed to the same altitude develops HAR. Candidate modifiers include ascent profile, exercise, carbon dioxide response, blood pressure, hematocrit, prior hemorrhage, vascular disease and genetic adaptation. Sex and hormonal status are underreported. Sleep-related hypoxemia may be more relevant than a daytime saturation. A susceptibility model should combine physiology with ocular reserve, then undergo external validation.

### 8.3 Connect retina and brain without claiming equivalence

Paired retinal, cerebral and systemic measurements could determine when the eye reflects shared hypoxic stress and when it behaves independently. OCTA or disc measures should be evaluated as incremental biomarkers beyond symptoms and oxygenation, not as stand-alone HACE tests. The most informative studies will align retinal imaging with cerebral perfusion or barrier-related measures across time while retaining clinical neurological outcomes.

### 8.4 Replace pathway lists with perturbation

The direct HAR literature now contains HIF, ROS, ER stress, NLRP3, lactate, mitophagy and ferroptosis. Adding another differentially expressed pathway is unlikely to change practice. The priority is to perturb a proposed target in a defined cell, demonstrate target engagement at a measured exposure, rescue a functional phenotype and test whether the effect survives reoxygenation. Negative or phase-dependent results should be published; they may explain why broad antioxidants have not become retinal treatments.

### 8.5 Build shared data and source standards

Imaging segmentation, chamber metadata, exposure logs and chemical fingerprints should be deposited in reusable formats where consent and regulation permit. Reference images and analysis code would improve reproducibility. For botanical interventions, voucher information and chromatographic data should be linked to the biological experiment. A natural-product result that cannot be tied to a reproducible material is not a transferable finding.

## 9 Conclusion

Taken together, HAR is best defined by altitude-attributable barrier, structural or functional injury rather than by every reversible vascular response. Multimodal longitudinal measurements are needed because vascular compensation, neural dysfunction and recovery can be asynchronous.

Clinical care prioritizes stopping ascent, oxygen or descent when indicated, systemic assessment and complication-specific ophthalmic management; established rescue protocols remain appropriate in emergencies despite the absence of HAR-specific drug trials.

Natural products remain preclinical leads. Progress now depends on stage-appropriate chemistry and exposure data, cell-specific causal testing, independent replication and longitudinal retinal function; claim strength should match the evidence tier.

## References

[B1] Arjamaa O. Nikinmaa M. (2006). Oxygen-dependent diseases in the retina: role of hypoxia-inducible factors. Exp. Eye Res. 83 (3), 473–483. 10.1016/j.exer.2006.01.016 16750526

[B2] Bailey J. A. Kong M. D. Hou B. Exinor A. Bao C. Tsang S. H. (2026). High-altitude retinopathy with isolated ellipsoid zone loss: a case report. Doc. Ophthalmol. 152 (1), 91–96. 10.1007/s10633-025-10067-6 41491338

[B3] Barthelmes D. Bosch M. M. Merz T. M. Petrig B. L. Truffer F. Bloch K. E. (2011). Delayed appearance of high altitude retinal hemorrhages. PLoS One 6 (2), e11532. 10.1371/journal.pone.0011532 21379571 PMC3040733

[B4] Bosch M. M. Merz T. M. Barthelmes D. Petrig B. L. Truffer F. Bloch K. E. (2009). New insights into ocular blood flow at very high altitudes. J. Appl. Physiol. (1985) 106(2)**,** 454–460. 10.1152/japplphysiol.90904.2008 19057000

[B5] Bosch M. M. Barthelmes D. Landau K. (2012). High altitude retinal hemorrhages--an update. High. Alt. Med. Biol. 13 (4), 240–244. 10.1089/ham.2012.1077 23270439

[B6] Brinchmann-Hansen O. Myhre K. Sandvik L. (1989). Retinal vessel responses to exercise and hypoxia before and after high altitude acclimatisation. Eye (Lond) 3 (Pt 6), 768–776. 10.1038/eye.1989.120 2630361

[B7] De Abreu J. Paillisser A. Berquet F. Hogg S. Trucco E. Geiser M. H. (2025). Changes in retinal vasculature phenotype and choroidal blood flow at high altitude in healthy lowlanders. Invest. Ophthalmol. Vis. Sci. 66 (6), 50. 10.1167/iovs.66.6.50 PMC12178430 40521978

[B8] Du N. Ge K. Xi H. Liu S. Zhang W. (2026). Inflammatory stress disrupts retinal lactate homeostasis under high-altitude hypoxia. Exp. Eye Res. 270, 111114. 10.1016/j.exer.2026.111114 42276506

[B9] Ebner L. J. A. Samardzija M. Storti F. Todorova V. Karademir D. Behr J. (2021). Transcriptomic analysis of the mouse retina after acute and chronic normobaric and hypobaric hypoxia. Sci. Rep. 11 (1), 16666. 10.1038/s41598-021-96150-9 34404875 PMC8371159

[B10] Fischer M. D. Willmann G. Schatz A. Schommer K. Zhour A. Zrenner E. (2012). Structural and functional changes of the human macula during acute exposure to high altitude. PLoS One 7 (4), e36155. 10.1371/journal.pone.0036155 22558365 PMC3340355

[B11] Ge K. Gu Y. Xi H. Liu S. Wang Y. Yang L. (2026). Targeting P2X7R protects the retina against high-altitude hypoxia via NLRP3-Mediated pyroptosis suppression. Mol. Neurobiol. 63 (1), 529. 10.1007/s12035-026-05828-6 41903063

[B12] Gok M. Karaman S. Erdem B. (2022). Evaluation of macular and choroidal thickness in healthy residents living at high altitude. Indian J. Ophthalmol. 70 (5), 1650–1655. 10.4103/ijo.IJO_2079_21 35502044 PMC9332972

[B13] Han C. Zheng X. X. Zhang W. F. (2024). High altitude retinopathy: an overview and new insights. Travel Med. Infect. Dis. 58, 102689. 10.1016/j.tmaid.2024.102689 38295966

[B14] Han C. Li Y. Zheng X. Zhang J. Zhao X. Ge K. (2025a). Choroidal thickening and retinal dopamine increase in mice at high altitude. Exp. Eye Res. 251, 110241. 10.1016/j.exer.2025.110241 39814194

[B15] Han C. Li Y. Zheng X. Zhang X. Li G. Zhao L. (2025b). AQP4- and Kir4.1-Mediated müller cell oedema is involved in retinal injury induced by hypobaric hypoxia. Mol. Neurobiol. 62 (2), 2012–2022. 10.1007/s12035-024-04382-3 39060906

[B16] Han C. Li Y. Qiao J. Zheng X. Liu Q. Wang Z. (2026). Salidroside ameliorated high-altitude exposure induced retinal ganglion cell death by activating HIF-1α/BNIP3 signaling mediated mitophagy and inhibiting ferroptosis. Phytomedicine 159, 158580. 10.1016/j.phymed.2026.158580 42480230

[B17] Huang H. (2020). Pericyte-endothelial interactions in the retinal microvasculature. Int. J. Mol. Sci. 21 (19), 7413. 10.3390/ijms21197413 33049983 PMC7582747

[B18] Janáky M. Grósz A. Tóth E. Benedek K. Benedek G. (2007). Hypobaric hypoxia reduces the amplitude of oscillatory potentials in the human ERG. Doc. Ophthalmol. 114 (1), 45–51. 10.1007/s10633-006-9038-5 17211646

[B19] Kaur C. Foulds W. S. Ling E. A. (2008). Blood-retinal barrier in hypoxic ischaemic conditions: basic concepts, clinical features and management. Prog. Retin Eye Res. 27 (6), 622–647. 10.1016/j.preteyeres.2008.09.003 18940262

[B20] Kur J. Newman E. A. Chan-Ling T. (2012). Cellular and physiological mechanisms underlying blood flow regulation in the retina and choroid in health and disease. Prog. Retin Eye Res. 31 (5), 377–406. 10.1016/j.preteyeres.2012.04.004 22580107 PMC3418965

[B21] Li C. Z. Wang L. L. Huang K. Zheng L. (2012). Endoplasmic reticulum stress in retinal vascular degeneration: protective role of resveratrol. Investigative Ophthalmol. and Vis. Sci. 53 (6), 3241–3249. 10.1167/iovs.11-8406 22491413

[B22] Li Y. Wang Q. Chu C. Y. Liu S. (2020). Astaxanthin protects retinal ganglion cells from acute glaucoma via the Nrf2/HO-1 pathway. J. Chem. Neuroanat. 110, 101876. 10.1016/j.jchemneu.2020.101876 33129943

[B23] Li J. Liu W. Q. Wang Y. F. Liu A. Q. Yu S. X. Yu H. D. (2023). Salidroside inhibits ganglion cell apoptosis by suppressing the muller cell inflammatory response in diabetic retinopathy. Curr. Eye Res. 48 (8), 758–769. 10.1080/02713683.2023.2204208 37287123

[B24] Li W. L. Li K. Chang W. G. Shi H. Zhang W. X. Wang Z. (2024). 20(R)-ginsenoside Rg3 alleviates diabetic retinal injury in T2DM mice by attenuating ROS-mediated ER stress through the activation of the Nrf2/HO-1 axis. Phytomedicine 135, 156202. 10.1016/j.phymed.2024.156202 39579577

[B25] Li J. Kong Y. Wang P. Chen C. Liu J. Li X. (2025a). Fundus changes in healthy adults after high altitude exposure. Photodiagnosis Photodyn. Ther. 53, 104542. 10.1016/j.pdpdt.2025.104542 40031993

[B26] Li N. Luo Y. Liu X. Tan D. Li Z. (2025b). Rhodiola tangutica (maxim.) S. H. Fu protects blood-retinal barrier in hypoxia-induced retinal injury rats by down-regulating HIF-1α/eNOS/NO pathway. J. Ethnopharmacol. 353 (Pt A), 120325. 10.1016/j.jep.2025.120325 40714061

[B27] Li Y. Han C. Zhang J. Huang S. Zhao X. Zhu J. (2025c). Microglia activation and migration in retina during acute high-altitude exposure. Exp. Eye Res. 261, 110653. 10.1016/j.exer.2025.110653 40998079

[B28] Li N. Ma X. L. Jin Y. H. Yan H. Y. Yun Y. G. Tan D. Y. (2026). Salidroside suppresses hypoxia-induced ocular angiogenesis by modulating the acetylation of PFKFB3 protein in endothelial cells and regulating glycolytic activity. Toxicol. Appl. Pharmacol. 514, 117921. 10.1016/j.taap.2026.117921 42336299

[B29] Linsenmeier R. A. Zhang H. F. (2017). Retinal oxygen: from animals to humans. Prog. Retin Eye Res. 58, 115–151. 10.1016/j.preteyeres.2017.01.003 28109737 PMC5441959

[B30] Liu S. Du N. Ge K. Hu J. Zhang W. (2025). NMN supplementation inhibits endothelial cell ROS-mediated Src/Pi3k/Akt signaling pathway to protect high-altitude blood-retinal barrier. Invest. Ophthalmol. Vis. Sci. 66 (4), 51. 10.1167/iovs.66.4.51 40249604 PMC12013676

[B31] Luks A. M. Swenson E. R. Bärtsch P. (2017). Acute high-altitude sickness. Eur. Respir. Rev. 26 (143), 160096. 10.1183/16000617.0096-2016 28143879 PMC9488514

[B32] Luks A. M. Beidleman B. A. Freer L. Grissom C. K. Keyes L. E. McIntosh S. E. (2024). Wilderness medical society clinical practice guidelines for the prevention, diagnosis, and treatment of acute altitude illness: 2024 update. Wilderness and Environ. Med. 35 (1_Suppl. L), 2S–19S. 10.1016/j.wem.2023.05.013 37833187

[B33] Luo H. D. Zhuang J. J. Hu P. P. Ye W. Chen S. S. Pang Y. L. (2018). Resveratrol delays retinal ganglion cell loss and attenuates gliosis-related inflammation from ischemia-reperfusion injury. Investigative Ophthalmol. and Vis. Sci. 59 (10), 3879–3888. 10.1167/iovs.18-23806 30073348

[B34] Ma J. Niu H. Ma X. Han C. Qu Y. (2022). Effects of long-term high-altitude exposure on retinal and choroidal microcirculation. Graefes Arch. Clin. Exp. Ophthalmol. 260 (11), 3525–3532. 10.1007/s00417-022-05699-2 35678838

[B35] Ma J. Niu H. Han C. Qu Y. (2023). Quantify retinal structure in high-altitude residents with and without high altitude polycythemia. BMC Ophthalmol. 23 (1), 6. 10.1186/s12886-022-02674-7 PMC9811807 36597056

[B36] MacCormick I. J. Somner J. Morris D. S. MacGillivray T. J. Bourne R. R. Huang S. S. (2012). Retinal vessel tortuosity in response to hypobaric hypoxia. High. Alt. Med. Biol. 13 (4), 263–268. 10.1089/ham.2011.1097 23270443

[B37] Maekawa S. Sato K. Fujita K. Daigaku R. Tawarayama H. Murayama N. (2017). The neuroprotective effect of hesperidin in NMDA-induced retinal injury acts by suppressing oxidative stress and excessive calpain activation. Sci. Rep. 7, 6885. 10.1038/s41598-017-06969-4 28761134 PMC5537259

[B38] Maria V. Kumar Khanna S. Chaudhary R. Maheshwari S. (2024). A multicentric cross-sectional observational study to analyze the effects of moderate high altitude on ocular health. Beyoglu Eye J. 9 (1), 48–54. 10.14744/bej.2023.81557 PMC10944850 38504963

[B39] McFadden D. M. Houston C. S. Sutton J. R. Powles A. C. Gray G. W. Roberts R. S. (1981). High-altitude retinopathy. Jama 245 (6), 581–586. 10.1001/jama.1981.03310310023016 7452886

[B40] McLaughlin K. Roy S. Falla M. Strapazzon G. Luks A. M. Zafren K. (2026). Pharmacological prophylaxis and supplemental oxygen for unacclimatized rescuers at very high altitude: scoping review and 2025 joint recommendations of the international commission for Mountain emergency medicine and the international society for Mountain medicine. High Alt. Med. and Biol. 27 (1), 60–77. 10.1177/15578682251365931 40844506

[B41] Mesentier-Louro L. A. Shariati M. A. Dalal R. Camargo A. Kumar V. Shamskhou E. A. (2020). Systemic hypoxia led to little retinal neuronal loss and dramatic optic nerve glial response. Exp. Eye Res. 193, 107957. 10.1016/j.exer.2020.107957 32032627 PMC7673281

[B42] Müllner-Eidenböck A. Rainer G. Strenn K. Zidek T. (2000). High-altitude retinopathy and retinal vascular dysregulation. Eye (Lond) 14 (Pt 5), 724–729. 10.1038/eye.2000.192 11116693

[B43] Neumann T. Baertschi M. Vilser W. Drinda S. Franz M. Brückmann A. (2016). Retinal vessel regulation at high altitudes1. Clin. Hemorheol. Microcirc. 63 (3), 281–292. 10.3233/ch-162041 26890235

[B44] Newman E. A. (2013). Functional hyperemia and mechanisms of neurovascular coupling in the retinal vasculature. J. Cereb. Blood Flow. Metab. 33 (11), 1685–1695. 10.1038/jcbfm.2013.145 23963372 PMC3824187

[B45] O'Leary F. Campbell M. (2023). The blood-retina barrier in health and disease. Febs J. 290 (4), 878–891. 10.1111/febs.16330 34923749

[B46] Paillisser A. Brugniaux J. V. Berquet F. De Abreu J. Hogg S. Trucco E. (2026). Effect of chronic high-altitude hypoxia on retinal vascular phenotype, choroidal and retinal vasoreactivity in Peruvian highlanders. Exp. Physiol. 111 (10), 4814–4826. 10.1113/ep093967 PMC13476870 42603100

[B47] Pan L. Cho K. S. Yi I. To C. H. Chen D. F. Do C. W. (2021). Baicalein, baicalin, and wogonin: protective effects against ischemia-induced neurodegeneration in the brain and retina. Oxidative Med. Cell. Longev. 2021, 2021. 10.1155/2021/8377362 34306315 PMC8263226

[B48] Pang Y. L. Qin M. Q. Hu P. P. Ji K. B. Xiao R. H. Sun N. (2020). Resveratrol protects retinal ganglion cells against ischemia induced damage by increasing Opa1 expression. Int. J. Mol. Med. 46 (5), 1707–1720. 10.3892/ijmm.2020.4711 32901846 PMC7521588

[B49] Paracini M. Radwan A. Peron G. (2026). Food-derived bioactive compounds in ocular diseases: molecular mechanisms, clinical evidence, and translational challenges. J. Nutr. Biochem. 158, 110465. 10.1016/j.jnutbio.2026.110465 42448244

[B50] Pournaras C. J. Rungger-Brändle E. Riva C. E. Hardarson S. H. Stefansson E. (2008). Regulation of retinal blood flow in health and disease. Prog. Retin Eye Res. 27 (3), 284–330. 10.1016/j.preteyeres.2008.02.002 18448380

[B51] Rana V. Pannu A. Sadhukhan A. Bandopadhayay S. Kumar P. Kaushik J. (2025). Delayed high-altitude retinal hemorrhages in well-acclimatized lowlanders: a hidden risk unveiled. Eur. J. Ophthalmol. 35 (3), 1061–1066. 10.1177/11206721241292382 39428995

[B52] Ren Y. Liang H. Xie M. J. Zhang M. (2024). Natural plant medications for the treatment of retinal diseases: the blood-retinal barrier as a clue. Phytomedicine 130, 155568. 10.1016/j.phymed.2024.155568 38795692

[B53] Safarzadeh M. A. Baker J. R. Raj S. R. Alim U. Day T. A. Jendzjowsky N. G. (2026). Layer-specific choroidal vascular adaptation to high-altitude hypoxia revealed by functional OCT at 3,800 m. Funct. (Oxf) 7 (4), e0022026. 10.1152/function.002.2026 42233517 PMC13290071

[B54] Schatz A. Willmann G. Fischer M. D. Schommer K. Messias A. Zrenner E. (2013). Electroretinographic assessment of retinal function at high altitude. J. Appl. Physiol. (1985) 115(3)**,** 365–372. 10.1152/japplphysiol.00245.2013 23722709

[B55] Seth R. K. Adelman R. A. (2010). High-altitude retinopathy and optical coherence tomography findings. Semin. Ophthalmol. 25 (1-2), 13–15. 10.3109/08820538.2010.481560 20507191

[B56] Sheu S. J. Liu N. C. Ou C. C. Bee Y. S. Chen S. C. Lin H. C. (2013). Resveratrol stimulates mitochondrial bioenergetics to protect retinal pigment epithelial cells from oxidative damage. Investigative Ophthalmol. and Vis. Sci. 54 (9), 6426–6438. 10.1167/iovs.13-12024 24008411

[B57] Shi X. P. Liao S. Mi H. J. Guo C. R. Qi D. L. Li F. (2012). Hesperidin prevents retinal and plasma abnormalities in streptozotocin-induced diabetic rats. Molecules 17 (11), 12868–12881. 10.3390/molecules171112868 23117428 PMC6268103

[B58] Shi K. Wang X. L. Zhu J. Cao G. Q. Zhang K. Su Z. G. (2015). Salidroside protects retinal endothelial cells against hydrogen peroxide-induced injury via modulating oxidative status and apoptosis. Biosci. Biotechnol. Biochem. 79 (9), 1406–1413. 10.1080/09168451.2015.1038212 25921655

[B59] Shi X. Dong N. Qiu Q. Li S. Zhang J. (2021). Salidroside prevents hypoxia-induced human retinal microvascular endothelial cell damage Via miR-138/ROBO4 axis. Invest. Ophthalmol. Vis. Sci. 62 (9), 25. 10.1167/iovs.62.9.25 PMC8297420 34269814

[B60] Shi X. Li M. Zhang X. Yuan F. Liu Y. Lin J. (2025). Longitudinal assessment of retinal and visual pathway electrophysiology and structure after high altitude exposure. Graefes Arch. Clin. Exp. Ophthalmol. 263 (6), 1573–1583. 10.1007/s00417-024-06729-x 39820514

[B61] Shortt A. J. Howell K. O'Brien C. McLoughlin P. (2004). Chronic systemic hypoxia causes intra-retinal angiogenesis. J. Anat. 205 (5), 349–356. 10.1111/j.0021-8782.2004.00351.x PMC1571355 15575883

[B62] Shushanyan R. A. Grigoryan A. V. Abgaryan T. A. Karapetyan A. F. (2026). Dexamethasone pretreatment attenuates hypobaric hypoxia-induced histomorphological alterations in the rat retina and primary visual cortex. Anat. Cell Biol. 10.5115/acb.26.107 42596728

[B63] Smith B. Wetherill N. Morris D. S. (2025). High-altitude retinopathy presenting as blue spots with prolonged recovery: a case report. High. Alt. Med. Biol. 26 (1), 99–101. 10.1089/ham.2024.0139 39912260

[B64] Su T. Gu C. Draga D. Zhou C. Lhamo T. Zheng Z. (2021). Integrative analysis of miRNA-mRNA network in high altitude retinopathy by bioinformatics analysis. Biosci. Rep. 41 (1), BSR20200776. 10.1042/bsr20200776 33393628 PMC7809558

[B65] Totou S. Karmiris E. Kanakis M. Gartaganis P. Petrou P. Kalogeropoulos C. (2023). Impact of flight and equivalent short-term high-altitude exposure on ocular structures and function. Med. Hypothesis Discov. Innov. Ophthalmol. 12 (3), 127–141. 10.51329/mehdiophthal1478 38476577 PMC10926315

[B66] Wang H. Ye Q. Huang S. Ge K. Han C. Xi H. (2026a). Time-resolved oxidative stress and apoptosis in murine retina under acute hypobaric hypoxia with parallel activation of the LIF-JAK-STAT3 axis. Mol. Neurobiol. 63 (1), 366. 10.1007/s12035-025-05639-1 41543620

[B67] Wang M. L. Wang M. L. Dong C. H. Chen Y. Y. Liu H. Y. Yao H. (2026b). Hesperidin-loaded nanoparticles attenuate pathological angiogenesis in oxygen-induced retinopathy by modulating the retinal immune microenvironment. Acs Biomaterials Sci. and Eng. 12 (3), 1826–1847. 10.1021/acsbiomaterials.5c01924 41741960

[B68] Westwood J. Mayhook-Walker I. Simpkins C. Darby-Smith A. Morris D. Normando E. (2024). Retinal vascular changes in response to hypoxia: a high-altitude expedition study. High. Alt. Med. Biol. 25 (1), 49–59. 10.1089/ham.2023.0084 38011631

[B69] Wiedman M. Tabin G. (1986). High-altitude retinal hemorrhage as a prognostic indicator in altitude illness. Int. Ophthalmol. Clin. 26 (2), 175–186. 10.1097/00004397-198602620-00010 3710724

[B70] Wiedman M. Tabin G. C. (1999). High-altitude retinopathy and altitude illness. Ophthalmology 106 (10), 1924–1926. 10.1016/s0161-6420(99)90402-5 10519586

[B71] Willmann G. Fischer M. D. Schatz A. Schommer K. Messias A. Zrenner E. (2011). Quantification of optic disc edema during exposure to high altitude shows no correlation to acute mountain sickness. PLoS One 6 (11), e27022. 10.1371/journal.pone.0027022 22069483 PMC3206056

[B72] Willmann G. Fischer M. D. Schatz A. Schommer K. Gekeler F. (2013). Retinal vessel leakage at high altitude. Jama 309 (21), 2210–2212. 10.1001/jama.2013.5550 23736726

[B73] Xie T. Chen X. Chen W. Huang S. Peng X. Tian L. (2021). Curcumin is a potential adjuvant to alleviates diabetic retinal injury via reducing oxidative stress and maintaining Nrf2 pathway homeostasis. Front. Pharmacol. 12, 796565. 10.3389/fphar.2021.796565 34955862 PMC8702852

[B74] Xie Z. Ying Q. Luo H. D. Qin M. Q. Pang Y. L. Hu H. J. (2023). Resveratrol alleviates retinal ischemia-reperfusion injury by inhibiting the NLRP3/Gasdermin D/Caspase-1/Interleukin-1β pyroptosis pathway. Investigative Ophthalmol. and Vis. Sci. 64 (15), 28. 10.1167/iovs.64.15.28 38133508 PMC10746937

[B75] Xin X. Rodrigues M. Umapathi M. Kashiwabuchi F. Ma T. Babapoor-Farrokhran S. (2013). Hypoxic retinal muller cells promote vascular permeability by HIF-1-dependent up-regulation of angiopoietin-like 4. Proc. Natl. Acad. Sci. U. S. A. 110 (36), E3425–E3434. 10.1073/pnas.1217091110 23959876 PMC3767527

[B76] Xin X. Dang H. Zhao X. Wang H. (2017). Effects of hypobaric hypoxia on rat retina and protective response of resveratrol to the stress. Int. J. Med. Sci. 14 (10), 943–950. 10.7150/ijms.19391 28924365 PMC5599917

[B77] Xin X. Li Y. Liu H. (2020). Hesperidin ameliorates hypobaric hypoxia-induced retinal impairment through activation of Nrf2/HO-1 pathway and inhibition of apoptosis. Sci. Rep. 10 (1), 19426. 10.1038/s41598-020-76156-5 33173100 PMC7655840

[B78] Yang Y. Han C. Sun Y. Zhao X. Chen Z. Zhao L. (2024). Effects of acute high-altitude exposure on morphology and function of retinal ganglion cell in mice. Invest. Ophthalmol. Vis. Sci. 65 (10), 19. 10.1167/iovs.65.10.19 39133471 PMC11323993

[B79] Yao F. Jiang X. Y. Qiu L. Peng Z. X. Zheng W. Ding L. X. (2022). Long-term oral administration of salidroside alleviates diabetic retinopathy in db/db mice. Front. Endocrinol. 13, 861452. 10.3389/fendo.2022.861452 35370972 PMC8966089

[B80] Yin Y. Liu D. Tian D. (2018). Salidroside prevents hydroperoxide-induced oxidative stress and apoptosis in retinal pigment epithelium cells. Exp. Ther. Med. 16 (3), 2363–2368. 10.3892/etm.2018.6494 30210588 PMC6122584

[B81] Yu X. Li J. Wang Y. Li X. Ding L. (2026a). Dynamic neurovascular adaptation of the retina during high-altitude hypoxia: integrated analysis of ERG and OCTA changes in healthy subjects. J. Appl. Physiol. (1985) 140(3)**,** 733–744. 10.1152/japplphysiol.00977.2025 41665522

[B82] Yu X. Li J. Wang Y. Zhou J. Li X. Ding L. (2026b). Dynamic changes in ocular and retinal function across acute hypobaric hypoxia. Life Sci. Space Res. (Amst) 48, 188–195. 10.1016/j.lssr.2025.11.009 41565399

[B83] Zając H. Gałka-Zielony A. Turno-Kręcicka A. Horoch-Łyszczarek E. (2026). High-altitude retinopathy with bilateral macular hemorrhage and brain edema. J. Vitr. Dis., 24741264261440754. 10.1177/24741264261440754 42051909 PMC13111535

[B84] Zhang L. C. Li N. Xu M. Chen J. L. He H. Liu J. (2024). Salidroside protects RGC from pyroptosis in diabetes-induced retinopathy associated with NLRP3, NFEZL2 and NGKB1, revealed by network pharmacology analysis and experimental validation. Eur. J. Med. Res. 29 (1), 60. 10.1186/s40001-023-01578-6 PMC10799395 38243268

[B85] Zhao L. E. Ling L. B. Lu J. Jiang F. Sun J. C. Zhang Z. (2023). Reactive oxygen species-responsive mitochondria-targeted liposomal quercetin attenuates retinal ischemia-reperfusion injury via regulating SIRT1/FOXO3A and p38 MAPK signaling pathways. Bioeng. and Transl. Med. 8 (3), e10460. 10.1002/btm2.10460 37206232 PMC10189480

[B86] Zheng X. X. Han C. Yang Y. Zhang W. F. (2025). Inhibition of ER stress mitigates hypobaric hypoxia-induced retinal damage: a simulated high-altitude study. Neuroscience 580, 181–193. 10.1016/j.neuroscience.2025.06.044 40544910

